# Microdomain formation is a general property of bacterial membrane proteins and induces heterogeneity of diffusion patterns

**DOI:** 10.1186/s12915-018-0561-0

**Published:** 2018-09-03

**Authors:** Daniella Lucena, Marco Mauri, Felix Schmidt, Bruno Eckhardt, Peter L. Graumann

**Affiliations:** 1 SYNMIKRO, LOEWE Center for Synthetic Microbiology, Marburg, Germany; 2 Fachbereich Chemie, Philipps-Universität Marburg, Marburg, Germany; 3 INRIA Grenoble - Rhône-Alpes, Montbonnot, France; 4 Fachbereich Physik, Philipps-Universität Marburg, Marburg, Germany

**Keywords:** *B*. *subtilis*, Membrane protein localization, Membrane dynamics, Diffusion, Single-molecule tracking

## Abstract

**Background:**

Proteins within the cytoplasmic membrane display distinct localization patterns and arrangements. While multiple models exist describing the dynamics of membrane proteins, to date, there have been few systematic studies, particularly in bacteria, to evaluate how protein size, number of transmembrane domains, and temperature affect their diffusion, and if conserved localization patterns exist.

**Results:**

We have used fluorescence microscopy, single-molecule tracking (SMT), and computer-aided visualization methods to obtain a better understanding of the three-dimensional organization of bacterial membrane proteins, using the model bacterium *Bacillus subtilis*. First, we carried out a systematic study of the localization of over 200 *B*. *subtilis* membrane proteins, tagged with monomeric mVenus-YFP at their original gene locus. Their subcellular localization could be discriminated in polar, septal, patchy, and punctate patterns. Almost 20% of membrane proteins specifically localized to the cell poles, and a vast majority of all proteins localized in distinct structures, which we term microdomains. Dynamics were analyzed for selected membrane proteins, using SMT. Diffusion coefficients of the analyzed transmembrane proteins did not correlate with protein molecular weight, but correlated inversely with the number of transmembrane helices, i.e., transmembrane radius. We observed that temperature can strongly influence diffusion on the membrane, in that upon growth temperature upshift, diffusion coefficients of membrane proteins increased and still correlated inversely to the number of transmembrane domains, following the Saffman–Delbrück relation.

**Conclusions:**

The vast majority of membrane proteins localized to distinct multimeric assemblies. Diffusion of membrane proteins can be suitably described by discriminating diffusion coefficients into two protein populations, one mobile and one immobile, the latter likely constituting microdomains. Our results show there is high heterogeneity and yet structural order in the cell membrane, and provide a roadmap for our understanding of membrane organization in prokaryotes.

**Electronic supplementary material:**

The online version of this article (10.1186/s12915-018-0561-0) contains supplementary material, which is available to authorized users.

## Background

The cytoplasmic membrane has remarkably intricate temporal and spatial organization that is central for the maintenance of fundamental biological processes such as cell division, endocytosis, morphogenesis, and chemotaxis [1]. The dynamic localization of membrane proteins constitutes an integral feature of membrane regulation, and lateral diffusion is critical for the modulation of shape and the distribution of proteins and lipids [2–4]. While initially conceived as a homogeneous lipid bilayer serving as a reaction platform for freely diffusing proteins, the cytoplasmic membrane is now widely accepted as being a highly dynamic and compartmentalized environment characterized by heterogeneous diffusion and the presence of microdomains, allowing lipids and proteins to be organized in specific regions of varying size and composition [2, 5–7]. Early models of the membrane often envisioned proteins as freely and homogeneously diffusing at all length scales, but this has long been known not to be the case in eukaryotic cells [8–10]. Many membrane proteins exhibit complex diffusive and anomalous behavior, reflecting the presence of structure in biological membranes [10, 11]. Membrane nanodomains enriched in cholesterol [12–17], protein–protein interactions [18], and interactions between transmembrane proteins and the cytoskeleton [17] have been all implicated in providing compartmentalization to the membrane [19, 20]. An emerging concept attributes the actin cytoskeleton the ability of imposing barriers or fences that restrict the lateral mobility of transmembrane proteins [19]. Fluctuations in the cytoskeleton allow the inter-compartmental barriers to be traversed by moving molecules, in a “hop-diffusion” manner [2, 16]. There is also growing evidence in support of lipid microdomains in bacteria [21–23]. For instance, cardiolipin was reported to be enriched in the polar regions of *Escherichia coli* [24], and small regions of high order, enriched in specific lipids that recruit and anchor a subset of membrane proteins, have been reported in *Bacillus subtilis* [3, 25]. Moreover, several *B*. *subtilis* membrane proteins have been shown to cluster into structures of 60 to 110 nm, supporting evidence for the existence of defined-size protein microdomains [26].

Ever since Singer and Nicolson introduced the fluid mosaic model of the plasma membrane [27], efforts have been made to understand the diffusion of proteins within the membrane environment. The hydrodynamic model of Saffman and Delbrück (SD) proposed that diffusion in cellular membranes was Brownian in nature and that diffusion coefficients would be determined solely by temperature and membrane viscosity [28], but investigations into membrane diffusion in intact eukaryotic cells revealed that proteins in the cell membrane exhibit very complex dynamics [5, 29] and their diffusive properties can change over a wide range of sizes and durations [2, 6, 7, 17]. Therefore, the paradigm for membrane substructure has changed to the concept of a compartmentalized fluid [6, 12, 30]. It is the interactions between diffusing proteins and the underlying membrane substructure that maintain the observed heterogeneity, as well as the observation that membrane proteins have dramatic drops in diffusion coefficients upon oligomerization or complex formation [17, 29, 30]. In turn, variations in temperature constitute a perturbation that influences physical processes underlying membrane motion in vivo*.* Complex formation between proteins and rearrangement of lipids in the membrane critically depends on temperature-driven diffusion. However, this is at least in part counteracted by substantial adaptive changes in the prokaryotic cell membrane that are induced by temperature changes. For instance, bacteria change the composition of their membrane fatty acids in order to maintain the membrane in a liquid crystalline phase, which is essential for its proper functionality [31].

Patterns of subcellular localization of proteins and their dynamics are often critical to understanding their activity. The advent of genetically encoded fluorescent reporters linked to powerful cell-imaging technologies has enabled accurate visualization of protein localization and in vivo tracking of protein movement [1]. In particular, single-molecule tracking (SMT) constitutes a powerful approach for characterizing the dynamic behaviors of proteins and protein complexes [21]. It has provided a new view of the bacterial cell as a dynamic system in which changes in protein localization over time orchestrate growth and differentiation. A wealth of SMT experiments in eukaryotes have completely changed our notion of the cell membrane and have highlighted the crucial role of cell membrane organization and dynamics regulating cellular function [2]. Single-molecule tracking of membrane proteins in particular can provide biophysical information on membrane organization, structure, and dynamics, and grant valuable insight into the interaction of membrane proteins with their surroundings. The dynamic subcellular distribution of proteins within the membrane has never been extensively examined in bacteria, despite the usefulness and importance of these data. Although a few reports have described membrane protein diffusion in bacteria [3, 26, 32], a systematic investigation on the localization and diffusion of membrane proteins is lacking. Also, to date, there has been no broad study to evaluate how protein size, number of transmembrane domains, and temperature affect the diffusion of membrane proteins. In this work, we characterize the effects of these factors on membrane protein diffusion and investigate the dynamics of membrane organization in live *B*. *subtilis* cells. We have undertaken a systematic study of the localization of over 200 *B*. *subtilis* membrane proteins, which accounts for about 20% of all membrane proteins in this organism. We defined a range of distinct patterns of localization and describe, by using SMT, the diffusion behavior of 25 selected proteins, 19 of which bear transmembrane domains. After assertion that anomalous diffusion was not observed, we computed diffusion coefficients by fitting cumulative distribution functions and corrected track projection for membrane curvature by introducing a mean-field approach. Diffusion coefficients of the analyzed transmembrane proteins do not correlate with protein molecular weight but decrease with increasing transmembrane radius. Moreover, diffusion is better described by discriminating two protein populations with distinct diffusion coefficients. Also, we observed that temperature can influence the organization of membrane proteins and significantly impact their dynamics in accordance with the Saffman–Delbrück theory. By combining single-molecule fluorescence microscopy with quantitative image analyses, we visualized *B*. *subtilis* membrane protein tracks in a realistic three-dimensional cell representation and were able to discriminate them according to movement distribution, for which we observed no directionality bias.

## Results

### A majority of *B*. *subtilis* membrane proteins localizes to defined structures within the membrane, termed “microdomains”

**Fig. 1:**
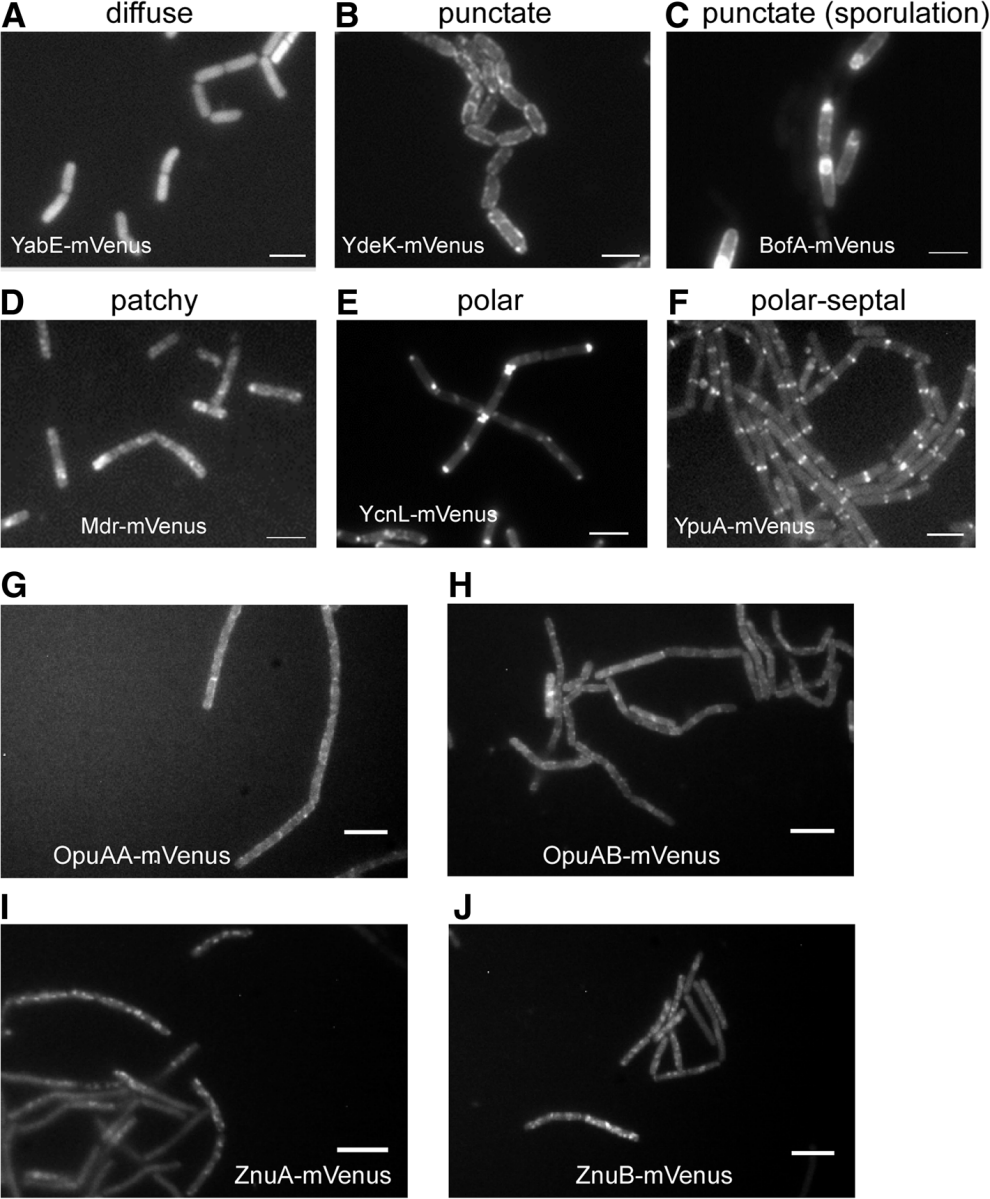
Representative pictures of fluorescence microscopy experiments showing patterns of membrane protein distribution according to their subcellular localization. The mVenus-tagged membrane proteins were observed in **a** diffuse, **b** punctate, **c** punctate/sporulation, **d** patchy, **e** polar, and **f** polar/septal patterns of distribution. **g**, **h** OpuAA and OpuAB form a complex in vivo, and both localize in a punctate pattern. **i**, **j** Both subunits of the ZnuAB complex localize in a patchy pattern. Gray lines in panels **a**–**f** 2 μm, white lines in **g**–**j** 3 μm

**Fig. 2:**
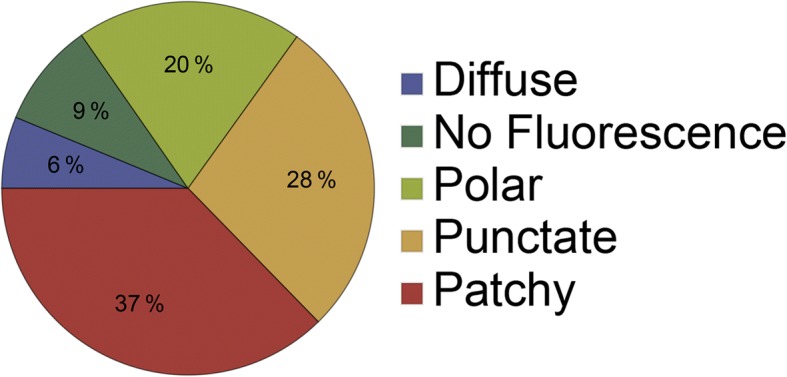
Pie chart representing membrane protein distribution according to their subcellular localization. For 15% of the proteins tested, there was no membrane localization, either diffuse (6%) or no fluorescence (9%) was observed. The remaining proteins were localized laterally on the membrane in a punctate fashion (28%), in a patchy distribution (37%), or at the cell poles and/or septa (20%)

To study the localization of membrane proteins, we constructed translational fusions of the gene for the Venus yellow fluorescent protein (Venus-YFP) to the 3′ end of genes coding for membrane proteins in the *B*. *subtilis* chromosome. The gene for the Venus YFP was modified by means of a mutation at amino acid 206 [33, 34] to render the fluorescent protein monomeric (which was then called mVenus). Cloning was performed with the plasmid pSG1164NLMV, a modified version of the pSG1164 vector [35] carrying a long and flexible linker coding for 15 amino acids (see Methods) between the gene of interest and the *mVenus* gene. Plasmid construction with primary cloning in *E. coli* was carried out in a high-throughput fashion with the Gibson Assembly technique [36]. Plasmids were integrated into the original gene locus on the *B*. *subtilis* chromosome, generating C-terminal membrane protein–mVenus fusions expressed under control of the original promoter as sole source of the protein in the cell. This strategy eliminates overproduction artifacts. *B*. *subtilis* cells carrying the fusions were analyzed during exponential and stationary phase, both in rich and in minimal medium. In total, we defined the subcellular localization of 209 different proteins (Figs. 1 and 2, Additional files 1 and 2). The patterns of fluorescence were classified with the aid of the microbe tracker software [37]; line scans were created to discriminate in an objective manner between the different fluorescence profiles (Additional file 1). Nine percent of the proteins showed no fluorescence (data not shown), meaning that the tagged protein was expressed below the detection threshold, or was not expressed at all, or was rapidly degraded. Six percent of the proteins showed diffuse cytoplasmic localization, indicating that the fusion was not functional and probably interfered with membrane insertion (Fig. 1a). Twenty-eight percent of the tagged proteins localized throughout the cell membrane wall and showed a finely punctate pattern within the membrane (Fig. 1b); 37% of the proteins localized throughout the membrane in a patchy fashion, where much larger structures could be seen (Fig. 1d) compared with the punctate patterns (Fig. 1b). The difference between patchy and punctate was also clearly visible in the line scans (Additional file 1). As a subclass of proteins localizing in a punctate pattern, BofA showed localization to the spores during late stationary phase (Fig. 1c), in agreement with its role as a sporulation protein [38]. Note that patchy localization could be caused by very large clusters of proteins, or by rapid diffusion during image acquisition, which would blur out defined-size microdomains. It is therefore possible that some of the patchy proteins actually belong to the punctate-pattern proteins. Interestingly, 20% of the fusion proteins localized predominantly to the cell poles (Fig. 1e, Fig. 2), which could be seen by characteristic peaks of fluorescence at the end of cells (Additional file 1). Therefore, a fifth of all membrane proteins prefers the cell pole for their subcellular localization. Protein YpuA localizes to the middle of cells, and to a minor degree to the cell poles (Fig. 1f), and is therefore a good candidate for a novel cell division protein.

We have compared all localizations from this study with previously published localization data, and all tested fusions exhibited fluorescent profiles similar to those previously described in the literature (e.g., ComN and MinJ as a polar proteins) [39–44], validating our approach. Also, proteins that have been reported to form complexes on the membrane showed similar localization patterns: subunits OpuAA and OpuAB of the osmolyte transporter OpuA localize in a similar punctate manner (Fig. 1g, h), while ZnuA and ZnuB form similar patchy structures within the membrane (Fig. 1i, j). The substitution of essential proteins by their fluorescently labeled versions (namely CdsA, FtsL, PbpB, PgsA, PlsC, PrsA, SecY, and WalK) indicates that the biological functions of the proteins were not significantly altered, and wild type-like proteins were produced. Altogether, our approach has provided positional information for 174 previously non-localized proteins and for 77 proteins of unknown function.

We addressed the question if microdomains formed by one membrane protein also contain other membrane proteins known to form a complex. We chose two pairs of proteins, YbaF and YuiG [45] and TatAC and TatCY [46]. mVenusYFP and CFP fusions of the complex partners indeed colocalized (Additional file 3), showing that microdomains can comprise several different proteins, in case of complex-forming membrane proteins.

These data show that there is a surprisingly high number of polar-localized proteins, clearly underlining the importance of these sites for the activity of membrane proteins (assuming that proteins are active where they are positioned), although poles may also serve as reservoirs for non-active proteins. Moreover, polar-localized proteins of unknown function, such as YbfB, YcnL, and YupA, constitute new candidate proteins that could play a role in cell division. The data further indicate that a vast majority of membrane proteins is present in clusters of several identical proteins within the cell membrane, because single fluorescent proteins cannot be detected using epifluorescence microscopy (and only by SMT, see below). It is still a matter of dispute if truly mixed, several protein species-containing microdomains exist in bacterial cells [22, 26, 47, 48], but clearly, most membrane proteins tend to stick together to at least their own kind.

### Transmembrane proteins exhibit heterogeneous diffusion

**Fig. 3:**
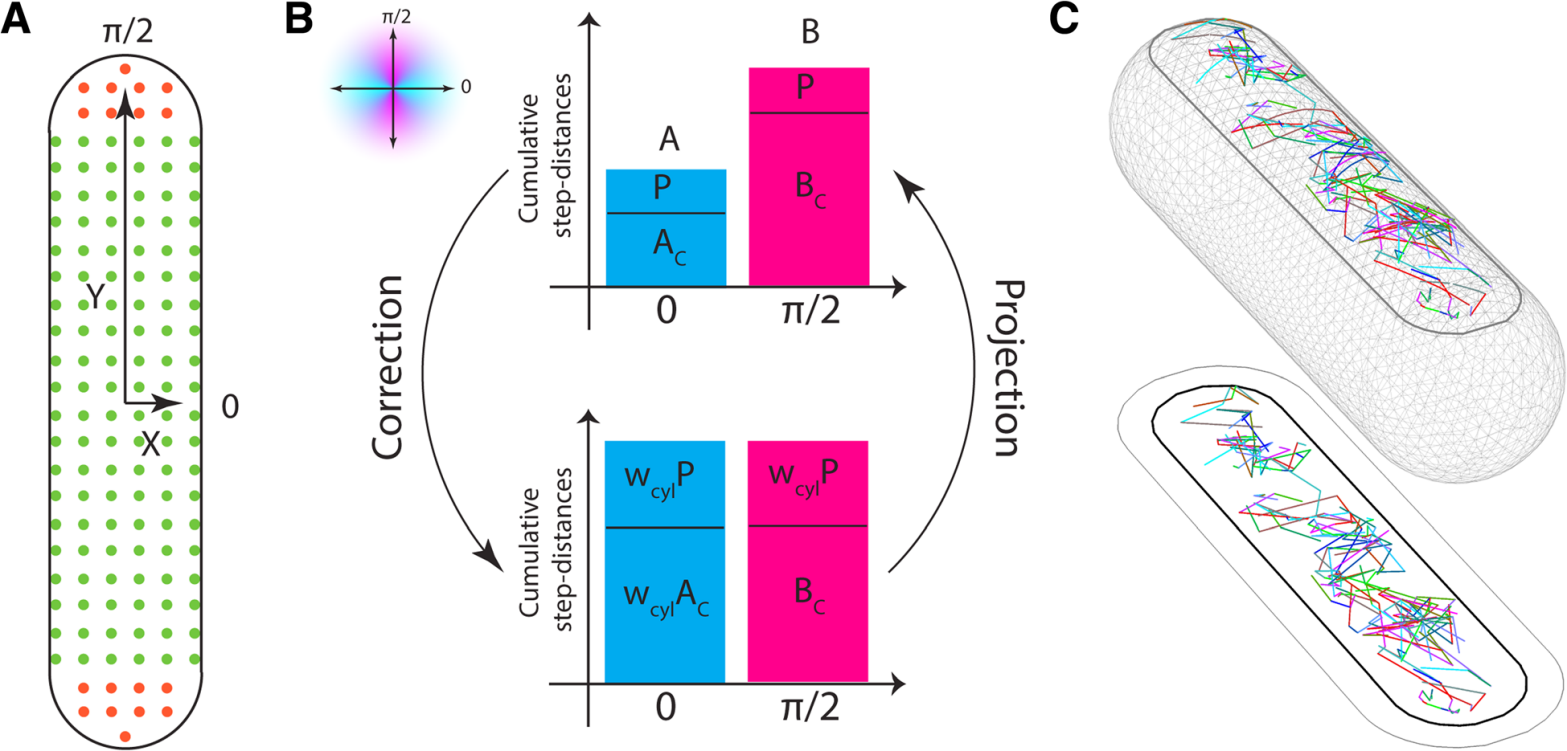
Curvature correction factor. In **a**, a schematic representation of a *B*. *subtilis* average cell aligned to coordinate axes. The polar and cylindrical regions are represented by orange and green, respectively. **b** Projection of curvature on observation plane impairs equality between components of cumulative step-distances along *x* (cyan) and *y* (magenta) axes. *P* (*P*) and *A*_*c*_ (*B*_*c*_) are the cumulative step-distances observed in the polar and cylindrical regions along short (long) axis. Equality can be restored by properly weighing these values by the factors *w*_cyl_ = *B*_*c*_/*A*_*c*_ and *w* = *B*/*A*, from which the curvature correction coefficient can be obtained (see Methods). **c** A realistic three-dimensional representation of tracks of the protein YbfF show that in our set-up, the curvature effect is not too pronounced. The region accessible with our depth of focus is delimited by the black curve. On the *xy* plane, we show the projections of observed boundary (gray), whole cell boundary (black), and the observed tracks

To gain deeper insight into membrane protein dynamics, we combined single-molecule tracking with cumulative distribution function (CDF) analysis for a subset of selected *B*. *subtilis* membrane proteins. For two-dimensional motion, CDF of the step sizes expresses the probability to find a protein inside a circle of radius *r*, given a localization accuracy *σ*, within a time lag *τ* [38]. Contrarily to averaging techniques, such as the mean square displacement (MSD) analysis, CDF can reveal heterogeneous diffusion of protein populations [47, 49] and by fitting, CDF identifies distinct diffusion coefficients for the subpopulations. Since proteins in SMT are difficult to follow for a long time, most of the tracks have few data-points. In our case, we chose four time lags as a good compromise between short tracks that suffer large relative localization error and long tracks with poor statistics [49, 50]. The explicit function used to describe the CDF curve depends on the specific diffusion model [51]. Here, we restricted the analysis to Brownian motion. This is mainly supported by the fact that a low number of time lags do not allow to properly discriminate whether diffusion is anomalous or not [51]. Examples for the mean squared displacement analyses are shown in Additional file 4

1 $$ \left\langle {r}^2\left(\tau \right)\right\rangle =4 D\tau +4{\sigma}^2 $$

where *D* is the diffusion coefficient and *σ* the localization error [47, 48]. Therefore, to obtain the diffusion coefficients, we fitted a function representing either one or more normally diffusing subpopulations to the CDF curve [3, 47]. The best fit was given by two populations with distinct diffusion coefficient *D*_1_ and *D*_2_

2 $$ P\left({r}^2,\tau \right)=1-\alpha \bullet \mathit{\exp}\left(\frac{-{r}^2}{4{D}_1\tau +4{\sigma}^2}\right)-\left(1-\alpha \right)\bullet \mathit{\exp}\left(\frac{-{r}^2}{4{D}_2\tau +4{\sigma}^2}\right) $$

and frequency *α* and (1 − *α*), respectively. Projection from a curved membrane to the microscope plane shrinks the short-axis component of tracks, leading to an underestimation of the diffusion coefficients. Several strategies have been developed to correct for the curvature such as probabilistic procedure [52], simulation [53], or analytical computation [3]. Here, we developed a mean-field method inspired by the approach of Dempwolff et al. [26]. The correction factor is computed from the dynamics of homogeneously distributed “calibration” proteins in cells for which the short and the long axes have been aligned to the *x* and *y* coordinate axes, respectively (Fig. 3, Additional file 5). Diffusion was decomposed into independent motions along the coordinate axes. In the absence of curvature, average quantities along both axes are equal. We explore the imbalance introduced by the reduction of distances along the short axis to obtain the correction factor

3 $$ C=\frac{{w_{\mathrm{cyl}}}^2}{2}\left(1+\frac{1}{w^2}\right) $$

where *w* and *w*_cyl_ are the ratios between the magnitude of cumulative distances projected along the *y* and *x* axes for the whole cell and only for the cylindrical region, respectively (Fig. 3). This correction can be applied to the diffusion coefficient of proteins in any randomly oriented cell, provided that tracks are not too spatially inhomogeneous. In our setup, the correction on the apparent diffusion coefficient was 23%. Final results for corrected diffusion coefficients of the two protein populations are reported in Table 1. We also found an average localization accuracy of *σ*^2^ = 6.3 ± 0.3 · 10^−3^μm^2^. Additional file 6 shows standard deviations for *D* (*D*_std_), *σ*^2^ (*σ*^2^_std_), and *α* (*α*_std_).

**Table 1:** Diffusion coefficients of 25 selected proteins of *B*. *subtilis* grown at 23 °C, 37 °C, or 43 °C

Protein	kDa	TMs	23 °C		37 °C		43 °C	
			μm^2^/s	% tracks	μm^2^/s	% tracks	μm^2^/s	% tracks
DivIVA	19.2	0/	0.00	53	0.01	39	0.00	41
		Associated*	0.37	47	0.60	61	0.63	59
EfeU	52.24	6	0.00	55	0.01	52	0.01	53
			0.34	45	0.41	48	0.42	47
FtsH	70.76	2	0.00	59	0.00	47	0.00	46
			0.55	41	0.82	53	0.85	54
GltP	44.45	12	0.00	57	0.00	70	0.00	74
			0.42	43	0.41	30	0.39	26
GuaB	52.82	0/	0.10	26	0.18	23	0.20	18
		Cytoplasmic	0.69	74	0.89	77	0.96	82
LmrB	51.54	14	0.00	55	0.00	57	0.00	60
			0.28	45	0.27	43	0.30	40
MinJ	43.51	7	0.00	60	0.00	45	0.00	46
			0.27	40	0.50	55	0.53	54
OpuAB	30.1	7	0.01	64	0.02	60	0.04	63
			0.44	36	0.42	40	0.46	37
YbaF	29.54	6	0.00	58	0.02	56	0.01	54
			0.31	42	0.44	44	0.49	46
YbfF	35.16	4	0.00	50	0.00	39	0.00	46
			0.37	50	0.65	61	0.68	54
YbxA	31.31	0/	0.00	58	0.04	55	0.04	59
		Associated	0.34	42	0.49	45	0.62	41
YceF	29.02	6	0.00	55	0.00	57	0.00	54
			0.50	45	0.59	43	0.63	46
YceJ	41.8	12	0.00	62	0.00	56	0.00	58
			0.18	38	0.26	44	0.22	42
Ycgq	33.06	4	0.00	58	0.01	55	0.01	62
			0.49	42	0.55	45	0.60	38
YcgR	31.78	8	0.00	54	0.00	64	0.01	59
			0.33	46	0.39	36	0.42	41
YciC	45.15	0/	0.00	53	0.01	44	0.01	39
		Associated	0.60	47	0.84	56	0.79	61
YdbL	12.79	4	0.01	63	0.02	51	0.02	45
			0.38	37	0.60	49	0.58	55
YddI	18.86	1	0.01	58	0.02	51	0.02	54
			0.50	42	0.80	49	0.71	46
YknZ	41.96	4	0.00	53	0.00	45	0.00	50
			0.22	47	0.57	55	0.68	50
YvcK	34.53	0/	0.02	48	0.05	42	0.06	43
		Cytoplasmic	0.59	52	0.97	58	0.90	57
YxcA	9.7	2	0.00	50	0.00	53	0.00	54
			0.23	50	0.58	47	0.60	46
YxeN	24.86	6	0.00	47	0.01	52	0.01	57
			0.28	53	0.39	48	0.47	43
YycG	69.86	2	0.01	47	0.00	42	0.00	43
			0.55	53	0.66	58	0.79	57
ZnuA	35.51	0/	0.01	63	0.01	57	0.02	58
		Lipid anchor	0.50	37	0.53	43	0.55	42
ZnuB	30.28	7	0.00	79	0.01	71	0.01	74
			0.42	21	0.49	29	0.47	26

Percentages of tracks show there are two different diffusing populations for each protein. Localization error in our system corresponds to a movement of 0.006 μm^2^/s, determined movement close to this number is stated as “0.00”. Standard deviations for all diffusions are shown in Additional file 6

*Membrane associated

**Fig. 4:**
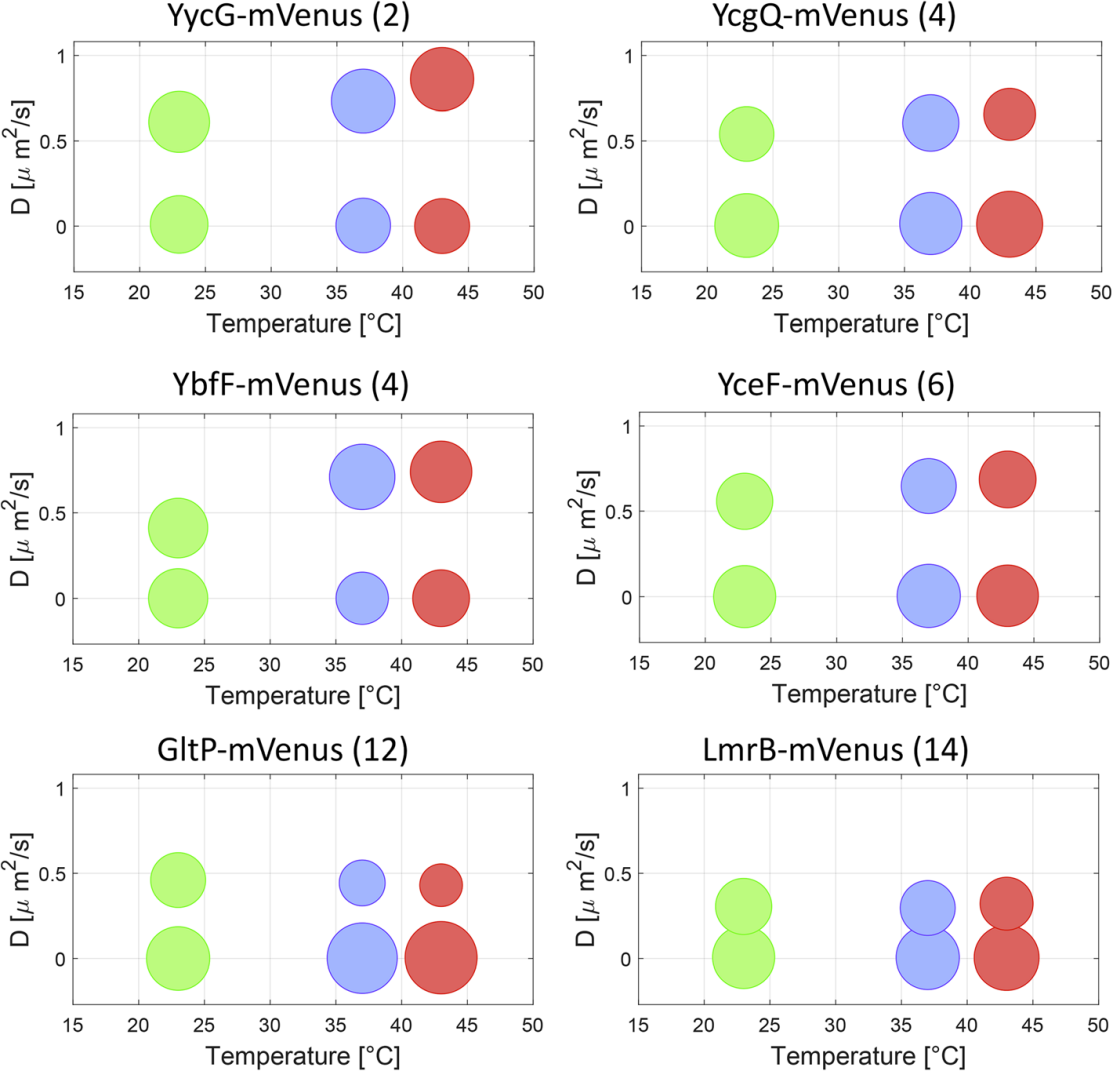
Variation of diffusion coefficients on dependence of growth temperature and number of transmembrane domains (shown in brackets). Graphs show two diffusing populations of mVenus-tagged proteins in *B*. *subtilis* PY 79 cultures grown at 23 °C, 37 °C, or 43 °C. The size of the circles corresponds to the percentage of molecules in each fraction. The number of transmembrane domains of each protein is given between parentheses

For all proteins analyzed, the CDF could be well fitted with the sum of two exponentials (see Additional file 7 for an example of the fitting), reflecting heterogeneous diffusion (Fig. 4). For example YycG-mVenus, YcgQ-mVenus, GltP-mVenus, and LmrB-mVenus, diffusion coefficients of the two populations differ by at least one order of magnitude (Table 1). The diffusion coefficients of the slow molecules suggest that these fractions are almost immobile (Fig. 4 and Additional file 8). The immobile fraction was also the one that accounted for a majority of the molecules. Separation in two different populations agrees well with the idea of membrane proteins moving together in microdomains (for a majority of the molecules), or independently of other proteins, as monomers (or dimers in case proteins form dimers as the smallest independent unit).

### 3D visualization and analyses of membrane protein diffusion reveal no directionality bias

**Fig. 5:**
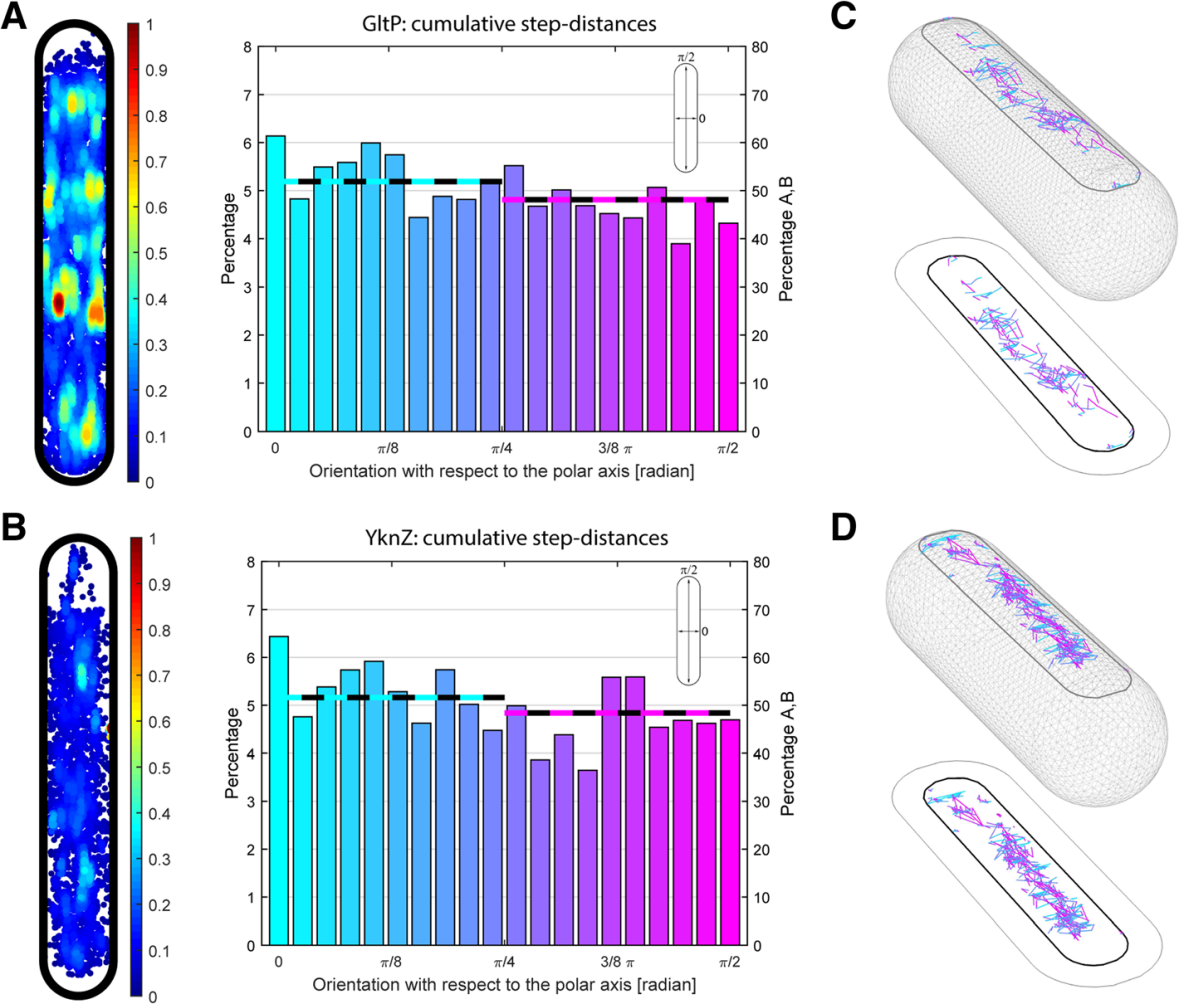
Density map and directionality histogram for proteins **a** GltP and **b** YknZ. The left panel shows density maps of the tracks in the average size of the bacterial cell from each protein database. In the histograms, the orientation of the tracks was calculated with respect to the short axis of the bacterial cells. The left *y* axis refers to the values of the cyan and magenta lines, which represent the cumulative step-distances along the short and long axis, respectively. Cumulative step-distances are the sum of all magnitudes of the components along the coordinate axes of all distances of every track at every time. The right *y* axis of the histograms shows the percentage for a finer division in smaller angles (20 bins). Distances were corrected for curvature as explained in the “Methods” section. **c**, **d** 3D visualization of tracks for two representative cells from the GltP and YknZ databases, respectively. The region accessible with our depth of focus is delimited by the black curve. Tracks are sorted according to their directions with the same color codes as in **a** and **b**

We wished to visualize tracks of membrane proteins on the bacterial wall surface in 3D and to analyze if movement may have a directional bias, e.g., possibly a preferential movement perpendicular to the long axis of the cells. This idea stems from the inhibition of filament formation of *E*. *coli* MreB protein, an actin-like protein that forms filaments directly underneath the cell membrane along the short axis of the cell, that was shown to increase diffusion coefficients of membrane proteins [3]. This finding implies that the filaments generate a diffusion barrier, which would disfavor diffusion along the long axis of the cell. To visualize tracks, we wrote a script in MATLAB to extract the track lengths from the raw data. Boundaries and central axes of the bacterium were used to create the 3D bacterial mesh surface with Wolfram Mathematica, taking into account that only tracks within our specific depth of focus were observed. Next, we computed, for each track, the analytical equation of the plane parallel to the *z* axis intersecting the *xy* surface. In that way, the planes of each track were intersected with the mesh surface to obtain the projection of the 2D tracks on the bacterial cell wall, generating realistic tracks that follow the geometry of the bacterial surface. Figure 3c shows YbfF tracks from several cells that have been adapted to an average cell, whereas Fig. 5c, d shows two single cell realizations from GltP and YknZ, respectively. By applying this analysis to every single cell, tracks could be visualized and discriminated according to the distance and direction traveled with respect to the short axis. We believe this tool is of use for all scientists investigating membrane protein dynamics.

We quantified how many tracks move along a specific direction by using the directionality histograms shown in Fig. 5. The cumulative density for GltP and YknZ, which display regions where they are more likely to be found, confirms they are punctate proteins. Correction for membrane curvature was performed with Eqs. 8 and 9 as described in the “Methods” section. The cumulative sum of the distance components along the *x* and *y* axes and a finer division 20-bin histogram were generated to reveal that, for all tested proteins, there is no orientation bias towards one specific direction (Fig. 5).

### Diffusion coefficients of transmembrane proteins do not correlate with molecular weight, but rather inversely with the number of transmembrane domains

**Fig. 6:**
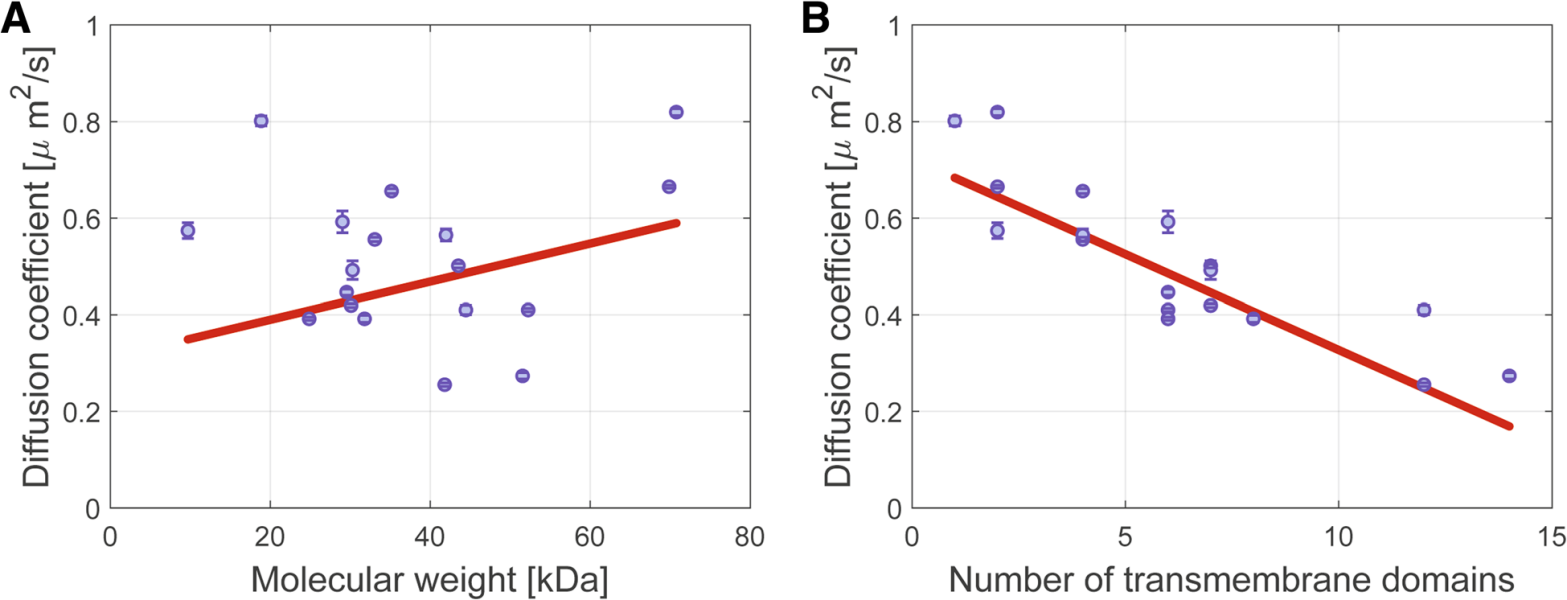
Dependence of membrane protein diffusion coefficients on protein size or on the number of transmembrane domains. Diffusion coefficients of mVenus fusion membrane proteins are plotted either **a** against protein molecular weight or **b** against the number of transmembrane domains. Linear trend lines are shown in red

Measuring and evaluating the mobility for 19 transmembrane proteins (Table 1) of varying molecular weights (from 10 to 70 kDa) and varying numbers of transmembrane domains (TMs) (from 1 to 14 TMs) allowed us to systematically test the dependence of the diffusion coefficient on the properties of selected proteins. The number of TMs can be considered as an indicator of the transmembrane radius. Because the slow-diffusing population of membrane proteins was almost immobile, we analyzed the faster-diffusing population of the selected membrane proteins. Plots of the diffusion coefficients (obtained from the CDF graphs from cells grown at 37 °C) against protein molecular weight showed no clear dependence of diffusion on molecular weight among the proteins tested (Fig. 6a), suggesting that protein molecular weight in itself is not a main determinant of membrane protein mobility. However, our set of transmembrane proteins with differently sized membrane inclusions allowed us to assess the dependence of diffusion on transmembrane insertions (Fig. 6b). To reveal the dependence on the number of TMs for diffusion in the *B*. *subtilis* cytoplasmic membrane, we plotted the diffusion coefficients against the number of TMs of the proteins and observed a decrease of the diffusion coefficient with higher number of TMs (Fig. 6b). The observed trend line reflects an inverse correlation between the number of transmembrane domains and the diffusion coefficient. Earlier models for membrane diffusion predict a dependence of the diffusion coefficient on the radius of the protein [28]. Our observations are consistent with the assumption that diffusion of proteins in the membrane should depend on the size of the membrane-spanning domain but not on the total protein size [54], and show that this is true for a large representative number of proteins. Outliers on the plots in Fig. 6b suggest a complexed state of smaller-radius proteins, which could have lower diffusion upon forming complexes with proteins that contain more TMs. Also, our data reveal that diffusion of membrane proteins is generally slower than that of cytosolic proteins (about four times slower than the cytosolic proteins we tested—Table 1), which had been seen in less systematic studies of membrane proteins [3, 11, 32].

### Upon temperature upshift, diffusion coefficients increase inversely proportional to the number of transmembrane domains

To our knowledge, the effect of temperature on the motion of proteins in the membrane has not been determined in a systematic manner. We therefore examined the influence of growth temperature on the diffusion of 25 selected proteins, including 19 transmembrane proteins (with the number of TMs varying from 1 to 14), one lipid-anchored protein, three membrane-associated proteins, and two cytoplasmic proteins that were added to the analysis for comparison (Table 1). To determine the dependence of diffusion on temperature, cells were grown at 23 °C, 37 °C, or 43 °C, and they were kept at the same temperature during single-molecule imaging. The proteins analyzed showed no substantial variation in their expression levels dependent on growth phase or temperature upshift [55]. As the cells were grown at higher temperatures, diffusion of some proteins increased significantly, even up to almost twofold (YycG, YbfF, Fig. 4). This result is qualitatively consistent with the assumption that at higher temperatures, there is increased protein motion. For YycG, with two TMs, there was an increase in the diffusion coefficient from 0.55 to 0.79 μm^2^/s in the mobile fraction, whereas the static fraction retained a low diffusion of 0–0.01 μm^2^/s (Fig. 4). Note that our localization precision on the diffusion coefficient is of 0.0063 μm^2^/s under the given imaging settings. Similar values were observed for YddI (1 TM) and FtsH (two TMs) (Additional file 8). For YcgQ (four TMs), YbfF (four TMs), and YceF (six TMs), an increase in the diffusion coefficient was also observed at different growth temperatures, mainly between 23 and 37 °C. However, only a minor increase in diffusion coefficients was observed for proteins bearing a high number of transmembrane domains (Fig. 4, Table 1). For GltP (12 TMs) and LmrB (14 TMs), the diffusion coefficients remained low and showed no significant changes, though the mobile fraction for GltP was reduced from 43% at 23 °C to 30% at 37 °C and 26% at 43 °C. The immobile population of LmrB at 43 °C was also slightly reduced, though the diffusion coefficients remained very similar at all three growth temperatures (Fig. 4).

**Fig. 7:**
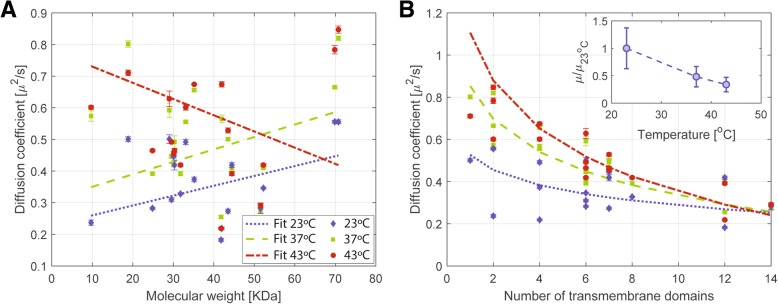
Dependence of membrane protein diffusion coefficients on temperature. Diffusion coefficients of selected mVenus fusion proteins were plotted against their molecular weight (**a**) and the number of transmembrane domains (TMs) (**b**). The data is fitted in **a** to a linear relation (for comparison) and in **b** to the Saffmann–Delbrück model. Dotted blue lines, dashed green lines, and dash-dotted red lines refer to 23 °C, 37 °C, and 43 °C, respectively. Inset in **b** shows the relative decrease of membrane viscosity as a function of temperature

To obtain deeper insight on the temperature effect, we analyzed how diffusion changed with temperature shifts depending either on the protein molecular weight or on the number of transmembrane domains. Figure 7 reveals that clearly, an increase in growth temperature does not lead to changes in transmembrane protein diffusion dependent on molecular weight, but rather a roughly inverse dependence on the number of transmembrane domains. For proteins with one to six TMs, the increase in diffusion coefficient at higher temperatures was much more remarkable than for proteins with a larger number of transmembrane domains (Fig. 4, Additional file 8). The Saffman–Delbrück theory predicts a logarithmic dependence of the protein’s diffusion coefficient on its hydrodynamic radius (here assumed to be proportional to the number of TMs) [29]. We fitted the diffusion coefficient curve versus the number of transmembrane domains (TM) at different temperatures to

4 $$ D=\frac{k_BT}{4\pi h{\mu}_m}\left(\ln \left(\frac{h{\mu}_m}{\mu {\prime}_{\mathrm{f}} TM}\right)-\gamma \right) $$

where *k*_B_ is the Boltzmann constant, *T* the absolute temperature, *h* the membrane thickness, *μ*_m_ the membrane viscosity, ln the natural logarithm, and γ Euler’s constant. *μ*′_f_ is an effective parameter defined as *nμ*_f_, with *μ*_f_ the viscosity of the surrounding fluid and *n* a conversion factor from the number of TMs to the actual hydrodynamic radius of the protein. Curves in Fig. 7b clearly show an increase in the diffusion coefficients upon rise in temperature, especially for proteins with smaller number of transmembrane domains. With a membrane thickness *h* of 2.7 nm [56, 57], we found a membrane viscosity of 1.16 ± 0.43Pa · s at 23 °C, 0.56 ± 0.06Pa · s at 37 °C, and 0.39 ± 0.04Pa · s at 43 °C in good agreement with values in the literature [58]. We also verified that the membrane viscosity decreases with the increasing temperature (inset of Fig. 7b) [59, 60]. The effective parameter *μ*′_f_ stays almost constant (within error range) over all temperatures. Moreover, with a few exceptions, most of the proteins analyzed did not show a significant variation in the percentages of mobile/immobile populations upon growth temperature upshift (Fig. 4 and Additional file 8). For membrane-associated and cytoplasmic proteins, an increase in the diffusion coefficients was also observed (Table 1). In general, a more striking increase in diffusion was observed between 23 and 37 °C (Fig. 4). The results fundamentally showed that growth temperature upshift had the general effect of increasing the diffusion coefficients with a dependence on the number of TMs and elicited no substantial change in the ratios of mobile/immobile populations.

## Discussion

In this work, we provide a detailed study on the localization and dynamics of a large number of membrane proteins in *B*. *subtilis*. To our knowledge, this is the first systematic study of spatiotemporal dynamics of bacterial membrane proteins. A majority of the more than 200 proteins analyzed localized in a punctate or patchy fashion, implying that multimerization, which can be due to protein/protein interactions and/or spatial proximity through specific lipid environment, is a major determinant for the spatial organization of membrane proteins. Our single-molecule tracking experiments performed with 25 selected proteins show that generally, a very slow-moving and a much more rapidly diffusing population can be identified for a given protein. This is in agreement with the formation of protein microdomains, and with monomers leaving these assemblies and diffusing freely, until a new assembly is met. Although several reports have indicated that many membrane proteins have a preferential localization to the cell poles, we were surprised that this is true for about 20% of all investigated proteins. Clearly, this subcellular region must confer specific advantages for the activity of a large fraction of membrane proteins, and although seen in many studies, our systemic approach unambiguously reveals the importance of this favored place of action in the membrane.

Also, we provide new biological insights through the localization of over 70 proteins with unknown functions (Additional file 2). The fast cloning approach with the Gibson assembly technique was very efficient and could be easily used to generate a collection of fluorescent fusions for the entire proteome of *B*. *subtilis* or for other bacteria in a high-throughput fashion. Another benefit of the employed approach is that upon plasmid integration in *B*. *subtilis*, the substitution of the gene’s original copy through its fluorescently labeled version is likely to eliminate any interference with the protein’s normal biological function, as well as any occurrence of overexpression artifacts often caused by heterologous expression. Besides, many of the fluorescent protein variants can form dimers at sufficiently high concentrations, an artifact that can perturb membrane structure or lead to incorrect assumptions when conducting quantitative advanced fluorescence techniques. The tagging of the proteins with monomeric Venus hinders the appearance of aggregation and oligomerization artifacts from the fluorescent protein itself.

Extending the systemic approach employed here, we furthermore demonstrate that the number of transmembrane domains or the radius of the membrane-spanning domains is indeed one of the main determinants for membrane protein mobility in *B*. *subtilis*, in contrast to cytoplasmic proteins where a dependence on molecular weight is found [32]. The observed dependence of mobility on the number of transmembrane domains shows that the membrane structure is dynamic and the fact that tracks can be discriminated into two diffusing populations agrees with the assumption that the cytoplasmic membrane is heterogeneous in its structure. Even very large membrane proteins with 14 transmembrane helices were found to have populations with faster and slower diffusion coefficients.

Other studies have resolved mixed populations of fast and slow diffusing species or found evidence of heterogeneous diffusion, which suggest structural order in bacterial membranes that may depend on length scale and location in the cell [11]. Our approach yields estimates for diffusion coefficients that are consistent with previously published values [11, 61–64]. There is likely not a sole but rather a combination of factors that could explain why membrane proteins have heterogeneous mobility and fit the CDF distribution into two diffusing populations. First, observations of halts and obstructions on single tracks could be caused by membrane proteins associated with the bacterial cytoskeleton. The effect of MreB on membrane organization has been observed for *B*. *subtilis* [25], and it has also been shown that the *E*. *coli* membrane protein diffusion is significantly faster upon inhibition of MreB polymerization [3]. The tendency for the formation of transient lipid domains in the absence of polymerized MreB indicates that lipid self-organization plays an important role in microdomain formation, similar to what has been proposed for the formation of eukaryotic lipid domains, and that MreB could stabilize them. For eukaryotic membranes, a fence model has been proposed, in which the cortical actin cytoskeleton forms compartments that physically restrict lipid diffusion [7]. Because the prokaryotic MreB cytoskeleton is less dense and also less coordinated, it is not clear if MreB can compartmentalize the prokaryotic membrane in the same way [3]. Flotilins have also been shown to be involved in setting up microdomains within the *B*. *subtilis* membrane, by recruiting other proteins and possibly specific lipids into special structures. Flotilin mutants also display altered membrane fluidity [65]. Membrane protein diffusion is therefore likely to be affected by microdomain formation, resulting in distinct diffusion coefficients in different regions of the membrane. Accordingly, the diffusion of almost all proteins investigated in this work could be well described by two different populations, supporting the assumption of bacterial membrane heterogeneity and regions of increased fluidity. Moreover, protein–protein interactions can also have a major effect on diffusion and even proteins with few transmembrane domains could have slower diffusion coefficients upon complex formation with proteins of a larger membrane radius.

By examining the temperature dependence of membrane protein dynamics in *B*. *subtilis*, we show that increased diffusion coefficients correlate with growth temperature upshift. Even though thermal fluctuations are probably not the only driving force for intracellular motion, we could observe that temperature has a significant effect in membrane protein diffusion. Membrane viscosity changes over threefold in the analyzed temperature range, leading to an increase in protein dynamics. ATP-dependent fluctuations can also contribute to macromolecular motion in vivo, in addition to thermal variations. An increase of ATP as a response to temperature upshift has already been described in *E*. *coli* [66]. Still, an increase in growth temperature had mitigated influence in the diffusion of proteins with a large number (more than six) of transmembrane domains, in accordance with the Saffman–Delbrück model. In our case, this model describes quite well the dependence of the diffusion coefficients on the number of transmembrane domains. It is known that under specific conditions, diffusion deviates from the canonical Saffman–Delbrück form, for instance in the presence of high molecular crowding [63], membrane inclusions larger than the SD-length scale [67], and large membrane protein assemblies [68, 69]. With our measurements, we cannot discriminate among these theories, but only confirm that our data agree with the basic relation describing logarithmic dependence of the diffusion coefficient on the number of transmembrane domain as in the SD theory.

Computer measurements of bacterial cells employed in this study provided very accurate visualization of tracks that follow the geometry of the bacterial surface. The idea that membrane proteins move in a restricted manner when diffusing laterally along the long axis of the cell is not genuine according to our results, as there is no preferred direction of diffusion and proteins moved rather randomly in all directions along the membrane. A recent study indicated that *B*. *subtilis* MreB shows no bias in its diffusion patterns as well [70]. Moreover, in this study, we provide a method to correct the diffusion coefficient for the membrane curvature. Indeed, projection of tracks to the microscope plane lowers apparent diffusion coefficients of proteins moving on curved membranes. Under our experimental conditions, the correction factor to the natural diffusion coefficient is 1.23.

## Conclusions

Our findings show that the membrane proteins of *B. subtilis* are generally organized into visually distinct microdomains. Because all of the 19 membrane proteins analyzed using SMT fall into two populations of low and high diffusion coefficients, individual proteins (or defined multimers thereof) freely diffuse, while microdomains consist of multimeric fractions. If microdomains consist of mixtures of different proteins, or of mostly individual proteins or protein complexes, remains to be investigated. Our results show that the number of transmembrane domains constitutes a major pacesetter of protein dislocation in the membrane and that temperature has a considerable influence on protein dynamics, especially for proteins with fewer TMs. The presence of distinct membrane microdomains and influence of temperature variations will have a strong impact on the spatiotemporal organization and thus function of the cell membrane.

## Methods

### Bacterial strains and plasmids

The membrane proteins [71, 72] analyzed in this study are listed in Additional file 2. Fusion genes with monomeric Venus YFP (mVenus) were cloned into plasmid pDL-mVenus, an expression plasmid containing the in-frame *mVenus* gene and a flexible 15-amino acid linker (GPGLSGLGGGGGSLG) downstream from the multiple cloning site. pDL-mVenus is a modified version of pSG1164 [35] that allows for native expression of genes of interest fused to genes coding for fluorescent proteins. Venus YFP was rendered monomeric by mutating amino acid 206 from lysine to arginine (A206K) [33, 34] using the quikchange mutagenesis kit (Agilent Genomics). The new linker was created by SOEing PCR with primers 5′CCTCCCAGGCCAGATAGGCCGGGCC3′ and 5′AAGGAGATTCCTAGGATGGGTACCG3′ downstream the xylose promoter of plasmid pSG1164 [35, 73]. Cloning was performed by using methods and reagents from the Gibson Assembly cloning system (New England Biolabs—NEB). A total of 500 pairs of PCR primers were designed to clone 500nt or less of ORFs of selected *B*. *subtilis* membrane protein-coding genes. Each forward and reverse primer contained sequences GATTCCTAGGATGGGTACCGGA and CAGGCCAGCCGGGCCC overlapping to the pDLmVenus vector, which was digested with *Apa*I and *Eco*RI restriction enzymes. PCR amplification was performed with Phusion polymerase (NEB) by following the manufacturer’s instructions, and *B*. *subtilis* wild-type strain PY79 chromosomal DNA was used as a template. The amplified gene product and the digested plasmid fragments were fused together by Gibson Assembly (60 min at 50 °C), using 1.33X Gibson master mix (NEB). After primary cloning in *E*. *coli* DH5α cells followed by ampicillin selection (100 μg/ml), PCR was performed by using specific primers flanking the insert region to verify the correct size of the inserted fragment. Plasmids from the *E*. *coli* clone library were then isolated using a mini-prep kit (Qiagen). *B*. *subtilis* PY79 or BG214 cells were transformed with the constructs in a high-throughput fashion, with selection for cloramphenicol resistance (5 μg/ml). For all proteins, mVenus was fused to the C-terminus and proteins were visualized as an mVenus fusion protein expressed at the original locus.

### Fluorescence microscopy and image classification

*B*. *subtilis* cultures were grown at 30 °C in LB broth or S750 minimal medium [74] until they reached stationary phase. Five milligrams per liter of cloramphenicol (Cm) were added to the media for selection. To ensure continuous nutrition supply and to immobilize the cells, 4 μl of cell culture were spotted on a cover slip and covered with a pad consisting of S750 minimal medium and 1% (*w*/*v*) agarose. Epifluorescence microscopy was done using a Zeiss Axio Imager A1 equipped with an EVOLVE EMCCD camera (Photometrics) and a TIRF objective with an aperture of 1.45, acquiring images with VisiView (2.1.2, Visitron, Munich) software and using a 515-nm laser for YFP detection. The resulting images were classified using MicrobeTracker [37]. Cell outlines were identified, cell length was measured, and intensity profiles were calculated for each cell (Additional file 1). Fusions from selected strains containing localized proteins were confirmed by sequencing.

### Single-molecule tracking

For single-molecule microscopy, a Nikon Eclipse Ti with a TIRF objective (× 100, Apo, NA: 1.49) was used. Image acquisition was accomplished using a back-illuminated EMCCD camera (Hamamatsu). The center of a 20-fold expanded beam from a 100-mW multiline argon laser (JDS Uniphase, laser head: 2219-G5MLS) was focused on the back focal plane and operated during image acquisition with 150 to 200 W/cm^2^. A 514-nm laser diode was used as excitation source. For image acquisition, the program Andor Solis 4.21 was applied. Streams of 1500 frames of 20 ms were acquired. Cells continued to grow after imaging, showing that there is little to no photo damage during acquisition. Acquired streams were loaded into Fiji ImageJ [75], and pixel sizes (106 nm) and time increments were calibrated. Tracking of single molecules was achieved using u-track 2.0 [76]. Only trajectories with at least four frames were used for further analysis to calculate the diffusion coefficients. Trajectory *x*/*y* coordinates and diffusion coefficients were calculated using a custom-made MATLAB script which included localization error. At least 1000 tracks were used for each analysis so that the CDF curves had at least 10^4^ points.

### Curvature correction

A membrane protein homogeneously distributed over a flat cell surface and undergoing Brownian motion is supposed to move in any direction with the same probability and, on average, with same step-distance. Therefore, in a cell for which the short and long axis have been aligned to the *x* and *y* axis (Fig. 3a) and the track distances *d* have been decomposed along coordinates, the average of distance magnitudes is expected to have the same value for both axes at each time step Δ*t*: ⟨|*d*(*x*, *Δt*)|⟩ = ⟨|*d*(*y*, *Δt*)|⟩ (Fig. 3b, lower panel). By reducing short-axis distances, projection from a curved membrane to a plane impairs such equivalence, as schematically shown in the upper panel of Fig. 7b. We quantify this inhomogeneity by the ratio

5 $$ w=\frac{\sum_{\varDelta t}\left\langle \left|{d}_{\mathrm{app}}\left(y,\varDelta t\right)\right|\right\rangle }{\sum_{\varDelta t}\left\langle \left|{d}_{\mathrm{app}}\left(x,\varDelta t\right)\right|\right\rangle}\equiv \frac{B}{A} $$

where *d*_app_(*x*, *Δt*) is the component of the apparent distance at every single time step Δt along the coordinate axis. For convenience, we called these values for the *x* and *y* components *A* and *B*, respectively. We define *w*_*x*_ and *w*_*y*_ the mean-field corrections of the step-distance components along *x* and *y* axes by

6 $$ {\sum}_{\varDelta t}\left\langle \left|d\left(x,\varDelta t\right)\right|\right\rangle ={w}_x{\sum}_{\varDelta t}\left\langle \left|{d}_{\mathrm{app}}\left(x,\varDelta t\right)\right|\right\rangle $$

7 $$ {\sum}_{\varDelta t}\left\langle \left|d\left(y,\varDelta t\right)\right|\right\rangle ={w}_y{\sum}_{\varDelta t}\left\langle \left|{d}_{\mathrm{app}}\left(y,\varDelta t\right)\right|\right\rangle . $$

Curvature affects the cylindrical and polar regions differently: in the first, only *x* components are diminished; in the latter, by symmetry, *x* and *y* components are equally affected. We call the apparent cumulative step-distances along the short axis in the cylindrical and polar regions *A*_c_ and *A*_p_, respectively, and along the long axis *B*_c_ and *B*_p_. Therefore, by symmetry *A*_p_ = *B*_p_ = *P* (Fig. 3b, upper panel). Under projection, *B*_c_ is unaffected (Fig. 3b, lower panel). On average, the correction factor of the cumulative step-distances along the *x* axis in the cylindrical region is therefore *w*_cyl_ = *B*_c_/*A*_c_. Again, by symmetry, the *x* and *y* components in the polar region are both corrected multiplying *P* by *w*_cyl_ (Fig. 3b, lower panel). We have then, in Eq. 6, *w*_*x*_∑_*Δt*_⟨|*d*_app_(*x*, *Δt*)|⟩ = *w*_*x*_(*P* + *A*_*c*_), ∑_*Δt*_⟨|*d*(*x*, *Δt*)|⟩ = *w*_cyl_(*P* + *A*_*c*_) and in Eq. 7 *w*_*y*_∑_*Δt*_⟨|*d*_app_(*y*, *Δt*)|⟩ = *w*_*y*_(*P* + *B*_*c*_), ∑_*Δt*_⟨|*d*(*y*, *Δt*)|⟩ = *w*_cyl_*P* + *B*_*c*_. Substituting these formulae in Eqs. 6 and 7, the correction factors *w*_*x*_ and *w*_*y*_ read

8 $$ {w}_x={w}_{\mathrm{cyl}}\frac{P+{A}_c}{P+{A}_c}={w}_{\mathrm{cyl}} $$

9 $$ {w}_y=\frac{w_{\mathrm{cyl}}P+{B}_c}{P+{B}_c}=\frac{w_{\mathrm{cyl}}}{w}. $$

If Eqs. 8 and 9 represent the correction that applies on average to each singular step-distance, the correction to the apparent diffusion coefficients for cells randomly oriented is then

10 $$ D=\frac{w_x^2+{w}_y^2}{2}{D}_{\mathrm{app}}=\frac{w_{cyl}}{2}\left(1+\frac{1}{w^2}\right){D}_{\mathrm{app}}\equiv C{D}_{\mathrm{app}} $$

In order to sample all curved space, the correction coefficient must be obtained from “calibration” proteins that are homogeneously distributed over the whole membrane. To find best candidates, we analyzed the density of proteins over average cells. Taking into account the depth of focus of 0.125 μm, the polar regions contribute for less than 10% of the total correction factor. We implemented a script in Wolfram Mathematica to automatically reconstruct cell meshes and realistic tracks from microscopic data into three dimensions (Fig. 3c). Finally, density maps revealed that MinJ satisfies at best the request of spatial homogeneity (Additional file 5) and it was used as a calibration protein. Since the observed area does not present a very high curvature, we do not expect a strong correction factor. The correction coefficient *C* was indeed 1.23, corresponding to a correction of 23% on the diffusion coefficients computed from randomly oriented cells. If cells are aligned to the coordinate system, *w*_*x*_ and *w*_*y*_ represent the independent corrections to the *x* and *y* components of each track. *P* and *B*_*c*_ in Eq. 9 represent weights that estimate the total distance that each protein travels in a specific region. For correcting diffusion of rotated cells, *P* and *B*_*c*_ can be specific from every single dataset. In such a way, only the factor *w*_cyl_ is needed from the calibration protein. Thus, the only requirement that a calibration protein has to satisfy is to diffuse homogeneously along the short axis direction. We used Eqs. 8 and 9 to correct the apparent directionality histograms that describe how many tracks move along a specific direction (left panels of Fig. 5).

## Additional files

Additional file 1: **Figure S1.** Fluorescent profiles obtained with the MicrobeTracker software. Representative pictures of the classified fluorescent profiles are shown on the left panel. The MicrobeTracker graphics on the right panel illustrate the fluorescence distribution obtained for proteins that localized with diffuse, polar, patchy or punctate fluorescence patterns (TIF 191 kb)

Additional file 2: **Table S1.** Subcellular localization of *B. subtilis* membrane proteins fused to mVenus (DOCX 71 kb)

Additional file 3: **Figure S2.** Colocalization patterns of *B. subtilis* transmembrane proteins. Pairs of transmembrane proteins of *B. subtilis* PY79 fusioned to CFP and mVenus show colocalization of proteins in common clusters. *mVenus* fusions to *yuiG* (upper panel) and *tatCY* (lower panel) were performed at the original gene locus and CFP fusions to *ybaF* (upper panel) and *tatAC* (lower panel) were integrated at the alpha amylase *amyE* locus. The arrows indicate tangible colocalization, the scale bar is 2 μm (TIF 402 kb)

Additional file 4: **Figure S3.** Mean square displacement (MSD) for GltP, MinJ, YbfF and YknZ proteins. MSD was computed from the ensemble average, where GltP, MinJ, YbfF and YknZ dataset contained 1224, 396, 207 and 696 tracks, respectively. Error bars represent the standard deviation divided by the square root of the degrees of freedom. We used four time lags for a total delay of 80 ms. Red dashed lines show the fit to a function with a linear relation on time (Equation 1 in the main text) (TIF 127 kb)

Additional file 5: **Figure S4.** Density map (A) and directionality histograms (B,C) for MinJ. (A) Density map of tracks in the average size of the bacterial cell from the protein database. Even though it is a polar protein, MinJ tracks are homogeneously distributed. (B, C) Histograms: orientation of the tracks was calculated with respect to the short axis of the bacterial cells. The MinJ dataset contained 2066 step-distances. Left y-axis refers to the values of the cyan and magenta lines, which represent the cumulative step-distances along the short and long axis, called A and B, respectively. Cumulative step-distances are the sum of all magnitudes of the components along the coordinate axis of all distances of every track at every time. The left y-axis of the histograms shows the percentage for a finer division in smaller angles (20 bins). In B, we show the histogram for which distances have not been corrected for the curvature. In C, the same histograms corrected for the curvature as explained in the Methods section (TIF 183 kb)

Additional file 6: **Table S2.** Diffusion coefficients with percentage of diffusing populations for *B. subtilis* proteins. Standard deviations are shown for D (Dstd), *σ*^2^(*σ*^2^std) and α(αstd) from all analyzed proteins (DOCX 63 kb)

Additional file 7: **Figure S5.** Step length distribution (A) and Cumulative Distribution Function (B) for tracks of protein YknZ-mVenus. (A) Steps lengths of the tracks in x and y directions are shown on the left panel and steps for both x and y are shown in the right panel. (B) For the CDF curves, experimental data are shown in blue and best fits for different population models are shown in red. Residuals (differences between data and fits) are shown in black. The shallower the black curve, the better is the agreement between actual data and fits. The graph on the left describes a one-term model (a single population), the one on the right describes a two-term model with two populations. Models with two terms described the CDF data best (TIF 165 kb)

Additional file 8: **Figure S6.** Variation of diffusion rates on dependence of growth temperature and number of transmembrane domains. Graphs show two diffusing populations of mVenus-tagged proteins in *B. subtilis* PY 79 cultures grown at 23°, 37° or 43° Celsius degrees. The number of transmembrane domains of each protein is given between parentheses (PDF 354 kb)

## Abbreviations

CDF: Cumulative distribution function
MP: Membrane protein
MSD: Mean square displacement
SD: Saffman and Delbrück
SMT: Single-molecule tracking
TM: Transmembrane domain

## References

[1] Shapiro L, McAdams HH, Losick R (2009). Why and how bacteria localize proteins. Science.

[2] Manzo C, Garcia-Parajo MF (2015). A review of progress in single particle tracking: from methods to biophysical insights. Rep Prog Phys.

[3] Oswald F, Varadarajan A, Lill H, Peterman EJ, Bollen YJ (2016). MreB-dependent organization of the E. coli cytoplasmic membrane controls membrane protein diffusion. Biophys J.

[4] Jackson D, Wang X, Rudner DZ (2012). Spatio-temporal organization of replication in bacteria and eukaryotes (nucleoids and nuclei). Cold Spring Harb Perspect Biol.

[5] Hofling F, Franosch T (2013). Anomalous transport in the crowded world of biological cells. Rep Prog Phys.

[6] Kusumi A, Suzuki KG, Kasai RS, Ritchie K, Fujiwara TK (2011). Hierarchical mesoscale domain organization of the plasma membrane. Trends Biochem Sci.

[7] Goiko M, de Bruyn JR, Heit B (2016). Short-lived cages restrict protein diffusion in the plasma membrane. Sci Rep.

[8] Engelman DM (2005). Membranes are more mosaic than fluid. Nature.

[9] Jacobson K, Mouritsen OG, Anderson RG (2007). Lipid rafts: at a crossroad between cell biology and physics. Nat Cell Biol.

[10] Ramadurai S, Holt A, Krasnikov V, van den Bogaart G, Killian JA, Poolman B (2009). Lateral diffusion of membrane proteins. J Am Chem Soc.

[11] Chow D, Guo L, Gai F, Goulian M (2012). Fluorescence correlation spectroscopy measurements of the membrane protein TetA in Escherichia coli suggest rapid diffusion at short length scales. PLoS One.

[12] Garcia-Parajo MF, Cambi A, Torreno-Pina JA, Thompson N, Jacobson K (2014). Nanoclustering as a dominant feature of plasma membrane organization. J Cell Sci.

[13] Lingwood D, Simons K (2010). Lipid rafts as a membrane-organizing principle. Science.

[14] Klotzsch E, Schutz GJ (2013). A critical survey of methods to detect plasma membrane rafts. Philos Trans R Soc Lond Ser B Biol Sci.

[15] Suzuki KG, Kasai RS, Hirosawa KM, Nemoto YL, Ishibashi M, Miwa Y, Fujiwara TK, Kusumi A (2012). Transient GPI-anchored protein homodimers are units for raft organization and function. Nat Chem Biol.

[16] Fujiwara T, Ritchie K, Murakoshi H, Jacobson K, Kusumi A (2002). Phospholipids undergo hop diffusion in compartmentalized cell membrane. J Cell Biol.

[17] Kusumi A, Nakada C, Ritchie K, Murase K, Suzuki K, Murakoshi H, Kasai RS, Kondo J, Fujiwara T (2005). Paradigm shift of the plasma membrane concept from the two-dimensional continuum fluid to the partitioned fluid: high-speed single-molecule tracking of membrane molecules. Annu Rev Biophys Biomol Struct.

[18] Chung I, Akita R, Vandlen R, Toomre D, Schlessinger J, Mellman I (2010). Spatial control of EGF receptor activation by reversible dimerization on living cells. Nature.

[19] Torreno-Pina JA, Manzo C, Salio M, Aichinger MC, Oddone A, Lakadamyali M, Shepherd D, Besra GS, Cerundolo V, Garcia-Parajo MF (2016). The actin cytoskeleton modulates the activation of iNKT cells by segregating CD1d nanoclusters on antigen-presenting cells. Proc Natl Acad Sci U S A.

[20] Akin EJ, Sole L, Johnson B, Beheiry ME, Masson JB, Krapf D, Tamkun MM (2016). Single-molecule imaging of Nav1.6 on the surface of hippocampal neurons reveals somatic nanoclusters. Biophys J.

[21] Rudner DZ, Losick R (2010). Protein subcellular localization in bacteria. Cold Spring Harb Perspect Biol.

[22] Lopez D, Koch G (2017). Exploring functional membrane microdomains in bacteria: an overview. Curr Opin Microbiol.

[23] Matsumoto K, Kusaka J, Nishibori A, Hara H (2006). Lipid domains in bacterial membranes. Mol Microbiol.

[24] Renner LD, Weibel DB (2011). Cardiolipin microdomains localize to negatively curved regions of Escherichia coli membranes. Proc Natl Acad Sci U S A.

[25] Strahl H, Burmann F, Hamoen LW (2014). The actin homologue MreB organizes the bacterial cell membrane. Nat Commun.

[26] Dempwolff F, Schmidt FK, Hervas AB, Stroh A, Rosch TC, Riese CN, Dersch S, Heimerl T, Lucena D, Hulsbusch N (2016). Super resolution fluorescence microscopy and tracking of bacterial flotillin (Reggie) paralogs provide evidence for defined-sized protein microdomains within the bacterial membrane but absence of clusters containing detergent-resistant proteins. PLoS Genet.

[27] Singer SJ, Nicolson GL (1972). The fluid mosaic model of the structure of cell membranes. Science.

[28] Saffman PG, Delbruck M (1975). Brownian motion in biological membranes. Proc Natl Acad Sci U S A.

[29] Metzler R, Jeon JH, Cherstvy AG, Barkai E (2014). Anomalous diffusion models and their properties: non-stationarity, non-ergodicity, and ageing at the centenary of single particle tracking. Phys Chem Chem Phys.

[30] Robson A, Burrage K, Leake MC (2013). Inferring diffusion in single live cells at the single-molecule level. Philos Trans R Soc Lond Ser B Biol Sci.

[31] Beranova J, Jemiola-Rzeminska M, Elhottova D, Strzalka K, Konopasek I (2008). Metabolic control of the membrane fluidity in Bacillus subtilis during cold adaptation. Biochim Biophys Acta.

[32] Kumar M, Mommer MS, Sourjik V (2010). Mobility of cytoplasmic, membrane, and DNA-binding proteins in Escherichia coli. Biophys J.

[33] Landgraf D, Okumus B, Chien P, Baker TA, Paulsson J (2012). Segregation of molecules at cell division reveals native protein localization. Nat Methods.

[34] Zacharias DA (2002). Sticky caveats in an otherwise glowing report: oligomerizing fluorescent proteins and their use in cell biology. Sci STKE.

[35] Lewis PJ, Marston AL (1999). GFP vectors for controlled expression and dual labelling of protein fusions in Bacillus subtilis. Gene.

[36] Gibson DG (2011). Enzymatic assembly of overlapping DNA fragments. Methods Enzymol.

[37] Sliusarenko O, Heinritz J, Emonet T, Jacobs-Wagner C (2011). High-throughput, subpixel precision analysis of bacterial morphogenesis and intracellular spatio-temporal dynamics. Mol Microbiol.

[38] Ricca E, Cutting S, Losick R (1992). Characterization of bofA, a gene involved in intercompartmental regulation of pro-sigma K processing during sporulation in Bacillus subtilis. J Bacteriol.

[39] Meile JC, Wu LJ, Ehrlich SD, Errington J, Noirot P (2006). Systematic localisation of proteins fused to the green fluorescent protein in Bacillus subtilis: identification of new proteins at the DNA replication factory. Proteomics.

[40] Rubio A, Pogliano K (2004). Septal localization of forespore membrane proteins during engulfment in Bacillus subtilis. EMBO J.

[41] Katis VL, Harry EJ, Wake RG (1997). The Bacillus subtilis division protein DivIC is a highly abundant membrane-bound protein that localizes to the division site. Mol Microbiol.

[42] Gamba P, Hamoen LW, Daniel RA (2016). Cooperative recruitment of FtsW to the division site of Bacillus subtilis. Front Microbiol.

[43] Noone D, Botella E, Butler C, Hansen A, Jende I, Devine KM (2012). Signal perception by the secretion stress-responsive CssRS two-component system in Bacillus subtilis. J Bacteriol.

[44] Nishibori A, Kusaka J, Hara H, Umeda M, Matsumoto K (2005). Phosphatidylethanolamine domains and localization of phospholipid synthases in Bacillus subtilis membranes. J Bacteriol.

[45] Eitinger T, Rodionov DA, Grote M, Schneider E (2011). Canonical and ECF-type ATP-binding cassette importers in prokaryotes: diversity in modular organization and cellular functions. FEMS Microbiol Rev.

[46] Monteferrante CG, Baglieri J, Robinson C, van Dijl JM (2012). TatAc, the third TatA subunit of Bacillus subtilis, can form active twin-arginine translocases with the TatCd and TatCy subunits. Appl Environ Microbiol.

[47] Lopez D (2015). Molecular composition of functional microdomains in bacterial membranes. Chem Phys Lipids.

[48] Bramkamp M, Lopez D (2015). Exploring the existence of lipid rafts in bacteria. Microbiol Mol Biol Rev.

[49] Michalet X (2010). Mean square displacement analysis of single-particle trajectories with localization error: Brownian motion in an isotropic medium. Phys Rev E Stat Nonlinear Soft Matter Phys.

[50] Qian H, Sheetz MP, Elson EL (1991). Single particle tracking. Analysis of diffusion and flow in two-dimensional systems. Biophys J.

[51] Metzler R, Klafter J. The restaurant at the end of the random walk: recent developments in the description of anomalous transport by fractional dynamics. J Phys A-Math Gen. 2004;37(31):R161–208.

[52] Oswald F, L M Bank E, Bollen YJ, Peterman EJ: Imaging and quantification of trans-membrane protein diffusion in living bacteria. Phys Chem Chem Phys 2014, 16(25):12625–12634. 10.1039/c4cp00299g 24760126

[53] Haas BL, Matson JS, DiRita VJ, Biteen JS (2014). Imaging live cells at the nanometer-scale with single-molecule microscopy: obstacles and achievements in experiment optimization for microbiology. Molecules.

[54] Gambin Y, Lopez-Esparza R, Reffay M, Sierecki E, Gov NS, Genest M, Hodges RS, Urbach W (2006). Lateral mobility of proteins in liquid membranes revisited. Proc Natl Acad Sci U S A.

[55] Nicolas P, Mader U, Dervyn E, Rochat T, Leduc A, Pigeonneau N, Bidnenko E, Marchadier E, Hoebeke M, Aymerich S (2012). Condition-dependent transcriptome reveals high-level regulatory architecture in Bacillus subtilis. Science.

[56] Inda ME, Oliveira RG, de Mendoza D, Cybulski LE (2016). The single transmembrane segment of minimal sensor DesK senses temperature via a membrane-thickness caliper. J Bacteriol.

[57] Nickels JD, Chatterjee S, Stanley CB, Qian S, Cheng X, Myles DAA, Standaert RF, Elkins JG, Katsaras J (2017). The in vivo structure of biological membranes and evidence for lipid domains. PLoS Biol.

[58] Loison P, Hosny NA, Gervais P, Champion D, Kuimova MK, Perrier-Cornet JM (2013). Direct investigation of viscosity of an atypical inner membrane of Bacillus spores: a molecular rotor/FLIM study. Biochim Biophys Acta.

[59] van de Vossenberg JL, Driessen AJ, da Costa MS, Konings WN (1999). Homeostasis of the membrane proton permeability in Bacillus subtilis grown at different temperatures. Biochim Biophys Acta.

[60] Mika JT, Thompson AJ, Dent MR, Brooks NJ, Michiels J, Hofkens J, Kuimova MK (2016). Measuring the viscosity of the Escherichia coli plasma membrane using molecular rotors. Biophys J.

[61] Rudner DZ, Pan Q, Losick RM (2002). Evidence that subcellular localization of a bacterial membrane protein is achieved by diffusion and capture. Proc Natl Acad Sci U S A.

[62] Deich J, Judd EM, McAdams HH, Moerner WE (2004). Visualization of the movement of single histidine kinase molecules in live Caulobacter cells. Proc Natl Acad Sci U S A.

[63] Javanainen M, Martinez-Seara H, Metzler R, Vattulainen I (2017). Diffusion of integral membrane proteins in protein-rich membranes. J Phys Chem Lett.

[64] Ritchie K, Spector J (2007). Single molecule studies of molecular diffusion in cellular membranes: determining membrane structure. Biopolymers.

[65] Lee YH, Kingston AW, Helmann JD (2012). Glutamate dehydrogenase affects resistance to cell wall antibiotics in Bacillus subtilis. J Bacteriol.

[66] Weber SC, Spakowitz AJ, Theriot JA (2012). Nonthermal ATP-dependent fluctuations contribute to the in vivo motion of chromosomal loci. Proc Natl Acad Sci U S A.

[67] Cicuta P, Keller SL, Veatch SL (2007). Diffusion of liquid domains in lipid bilayer membranes. J Phys Chem B.

[68] Camley BA, Zhao Y, Li B, Levine H, Rappel WJ (2013). Periodic migration in a physical model of cells on micropatterns. Phys Rev Lett.

[69] Naji A, Levine AJ, Pincus PA (2007). Corrections to the Saffman-Delbruck mobility for membrane bound proteins. Biophys J.

[70] Billaudeau C, Chastanet A, Yao Z, Cornilleau C, Mirouze N, Fromion V, Carballido-Lopez R (2017). Contrasting mechanisms of growth in two model rod-shaped bacteria. Nat Commun.

[71] van Dijl JM, Dreisbach A, Buist G, Sibbald MJJB, Zweers JC, Skwark MJ, Tjalsma H. The ins and outs of the Bacillus subtilis membrane proteome. In: Graumann P, editor. Bacillus: cellular and molecular biology. Norfolk: Horizon Scientific Press; 2012. p. 287–330.

[72] Michna RH, Zhu B, Mader U, Stulke J (2016). SubtiWiki 2.0--an integrated database for the model organism Bacillus subtilis. Nucleic Acids Res.

[73] Horton RM (1995). PCR-mediated recombination and mutagenesis. SOEing together tailor-made genes. Mol Biotechnol.

[74] Jaacks KJ, Healy J, Losick R, Grossman AD (1989). Identification and characterization of genes controlled by the sporulation-regulatory gene spo0H in Bacillus subtilis. J Bacteriol.

[75] Schindelin J, Arganda-Carreras I, Frise E, Kaynig V, Longair M, Pietzsch T, Preibisch S, Rueden C, Saalfeld S, Schmid B (2012). Fiji: an open-source platform for biological-image analysis. Nat Methods.

[76] Jaqaman K, Loerke D, Mettlen M, Kuwata H, Grinstein S, Schmid SL, Danuser G (2008). Robust single-particle tracking in live-cell time-lapse sequences. Nat Methods.

