# Supplementary figures and images for: Microdomain formation is a general property of bacterial membrane proteins and induces heterogeneity of diffusion patterns
Source: BMC Biol. 2018 Sep 3;16:97. doi: 10.1186/s12915-018-0561-0 (PMC6120080; doi:10.1186/s12915-018-0561-0)

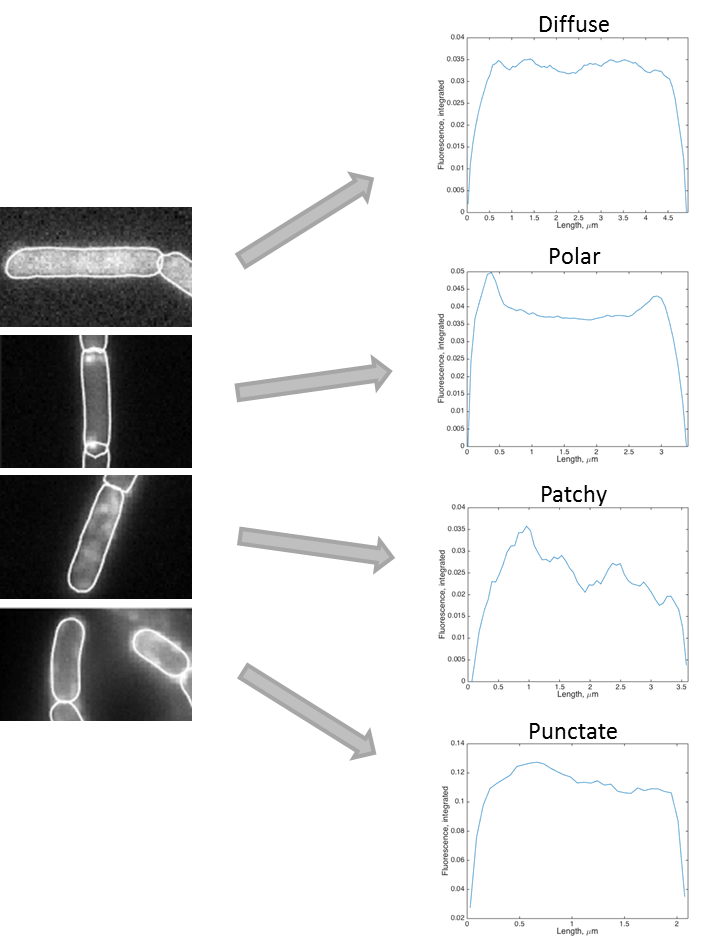

Supplement: Supplementary file 1 — Figure S1. Fluorescent profiles obtained with the MicrobeTracker software. Representative pictures of the classified fluorescent profiles are shown on the left panel. The MicrobeTracker graphics on the right panel illustrate the fluorescence distribution obtained for proteins that localized with diffuse, polar, patchy or punctate fluorescence patterns (TIF 191 kb) [file 12915_2018_561_MOESM1_ESM.tif]

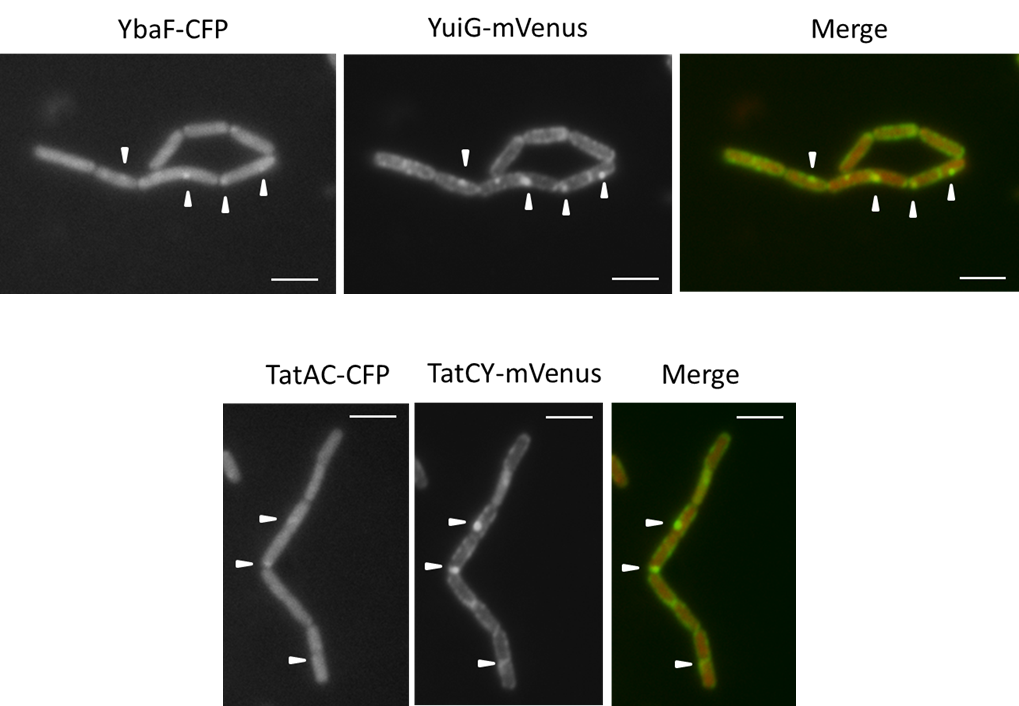

Supplement: Supplementary file 3 — Figure S2. Colocalization patterns of B. subtilis transmembrane proteins. Pairs of transmembrane proteins of B. subtilis PY79 fusioned to CFP and mVenus show colocalization of proteins in common clusters. mVenus fusions to yuiG (upper panel) and tatCY (lower panel) were performed at the original gene locus and CFP fusions to ybaF (upper panel) and tatAC (lower panel) were integrated at the alpha amylase amyE locus. The arrows indicate tangible colocalization, the scale bar is 2 μm (TIF 402 kb) [file 12915_2018_561_MOESM3_ESM.tif]

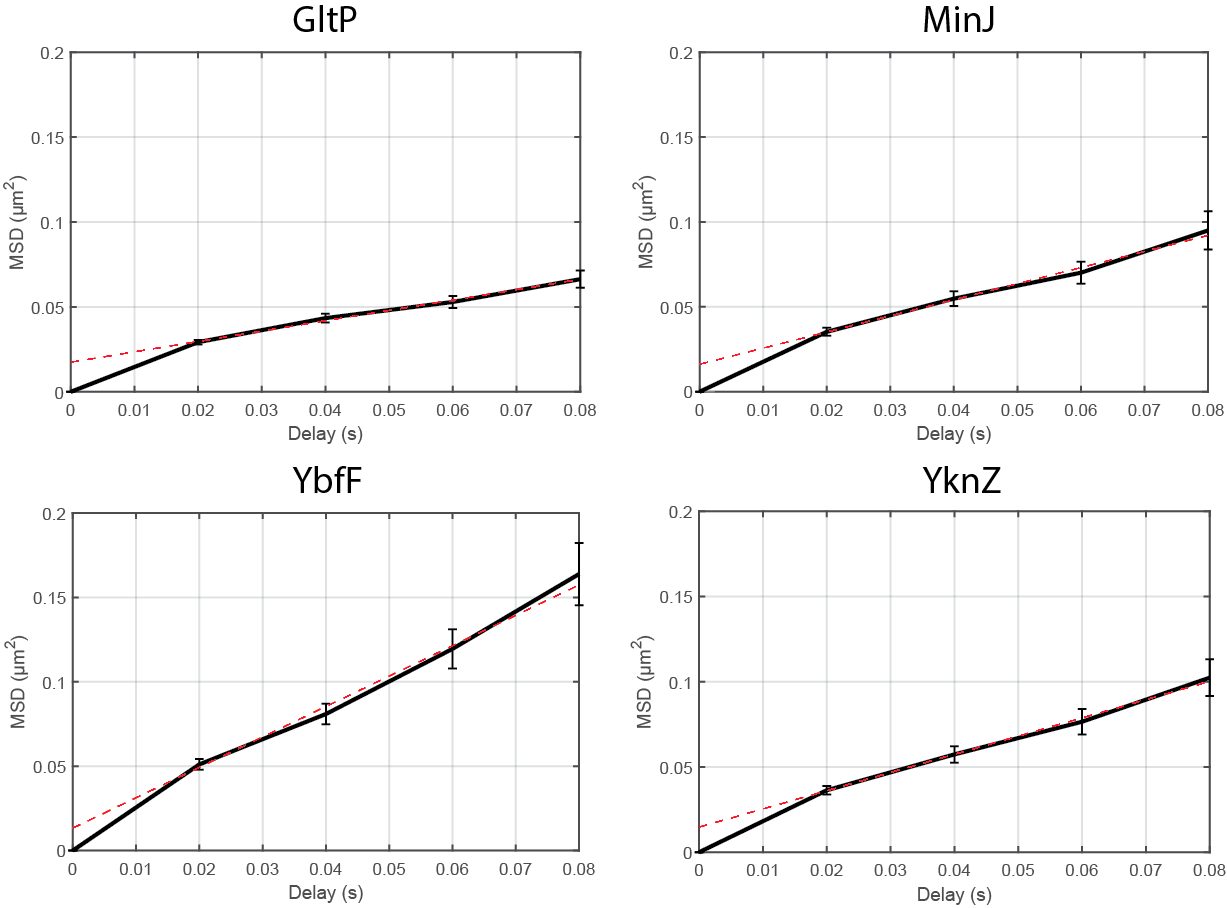

Supplement: Supplementary file 4 — Figure S3. Mean square displacement (MSD) for GltP, MinJ, YbfF and YknZ proteins. MSD was computed from the ensemble average, where GltP, MinJ, YbfF and YknZ dataset contained 1224, 396, 207 and 696 tracks, respectively. Error bars represent the standard deviation divided by the square root of the degrees of freedom. We used four time lags for a total delay of 80 ms. Red dashed lines show the fit to a function with a linear relation on time (Equation 1 in the main text) (TIF 127 kb) [file 12915_2018_561_MOESM4_ESM.tif]

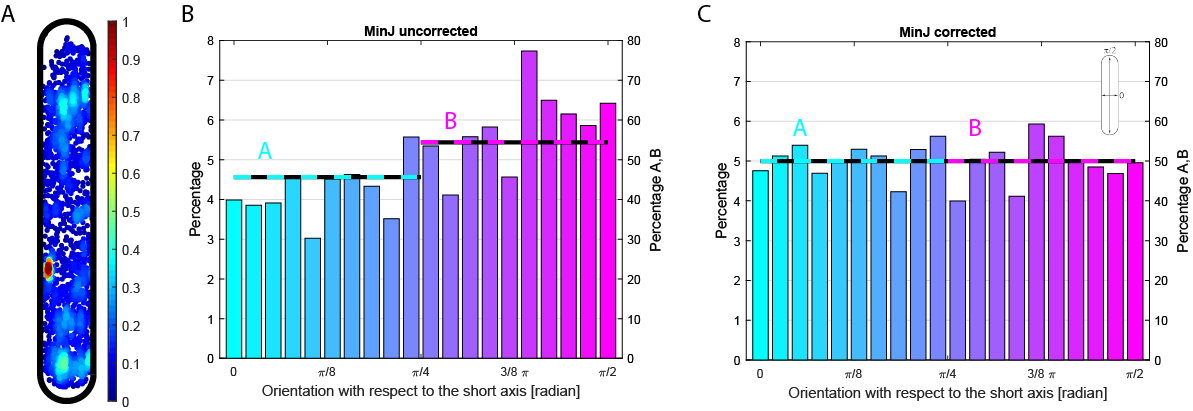

Supplement: Supplementary file 5 — Figure S4. Density map (A) and directionality histograms (B,C) for MinJ. (A) Density map of tracks in the average size of the bacterial cell from the protein database. Even though it is a polar protein, MinJ tracks are homogeneously distributed. (B, C) Histograms: orientation of the tracks was calculated with respect to the short axis of the bacterial cells. The MinJ dataset contained 2066 step-distances. Left y-axis refers to the values of the cyan and magenta lines, which represent the cumulative step-distances along the short and long axis, called A and B, respectively. Cumulative step-distances are the sum of all magnitudes of the components along the coordinate axis of all distances of every track at every time. The left y-axis of the histograms shows the percentage for a finer division in smaller angles (20 bins). In B, we show the histogram for which distances have not been corrected for the curvature. In C, the same histograms corrected for the curvature as explained in the Methods section (TIF 183 kb) [file 12915_2018_561_MOESM5_ESM.tif]

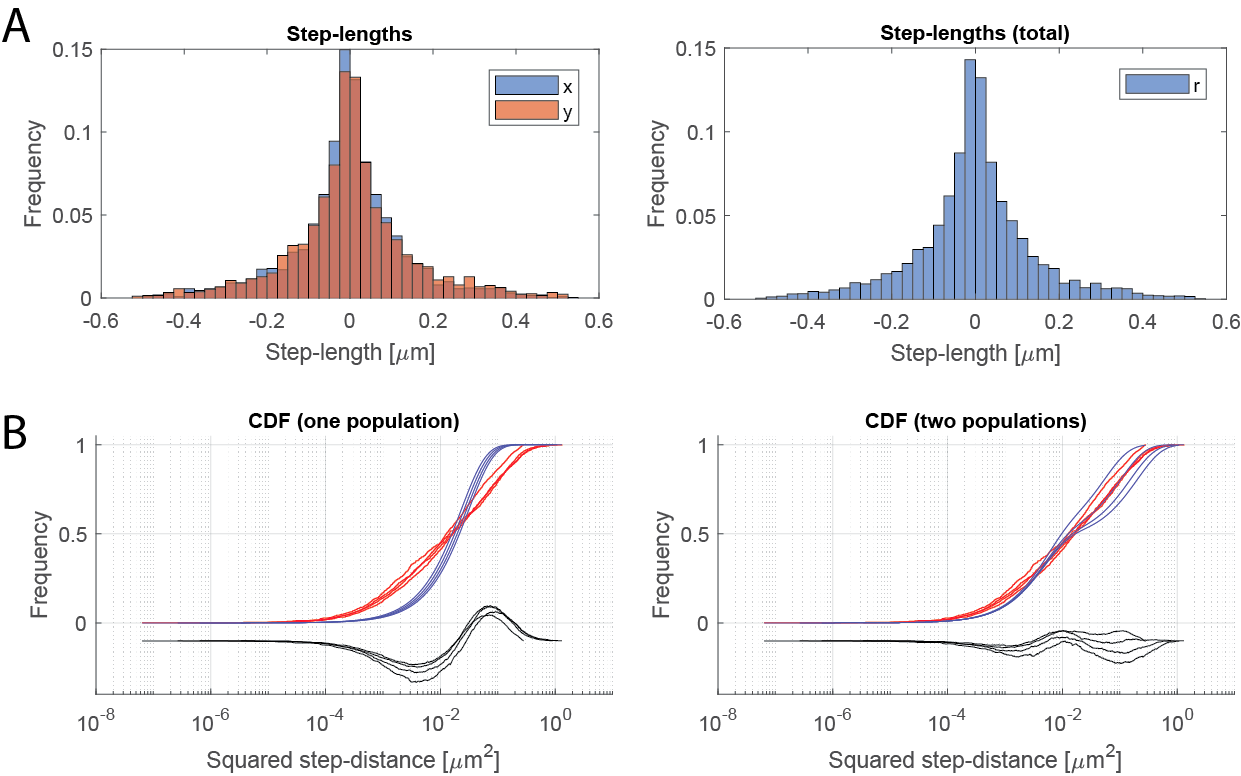

Supplement: Supplementary file 7 — Figure S5. Step length distribution (A) and Cumulative Distribution Function (B) for tracks of protein YknZ-mVenus. (A) Steps lengths of the tracks in x and y directions are shown on the left panel and steps for both x and y are shown in the right panel. (B) For the CDF curves, experimental data are shown in blue and best fits for different population models are shown in red. Residuals (differences between data and fits) are shown in black. The shallower the black curve, the better is the agreement between actual data and fits. The graph on the left describes a one-term model (a single population), the one on the right describes a two-term model with two populations. Models with two terms described the CDF data best (TIF 165 kb) [file 12915_2018_561_MOESM7_ESM.tif]

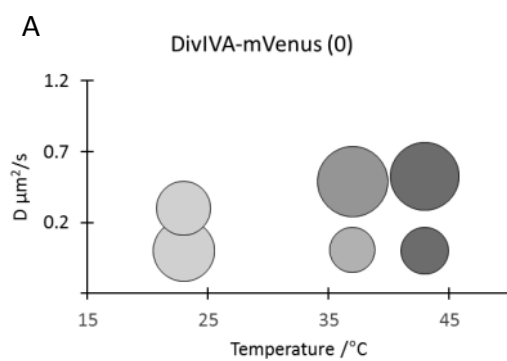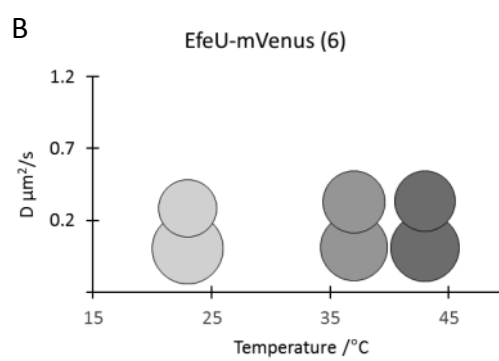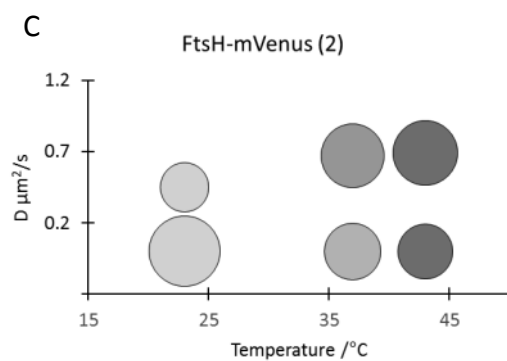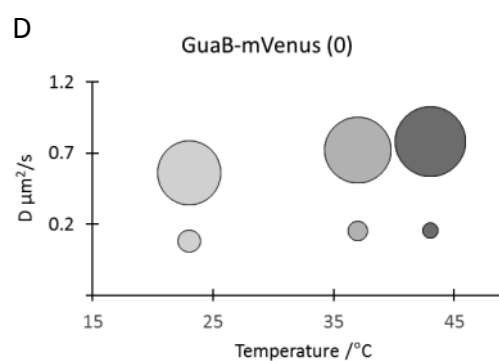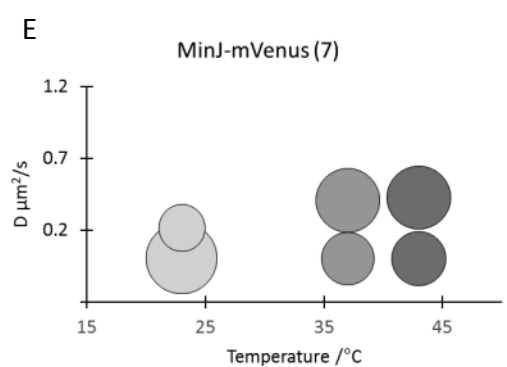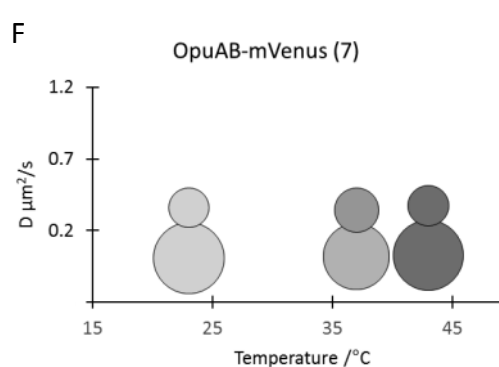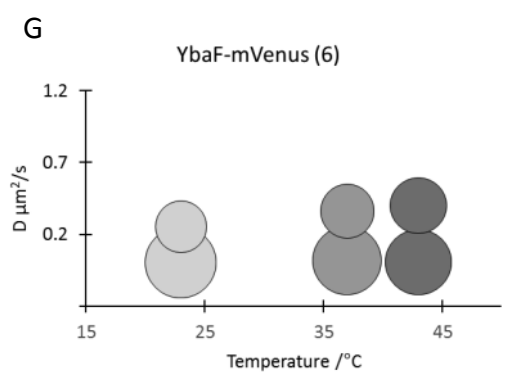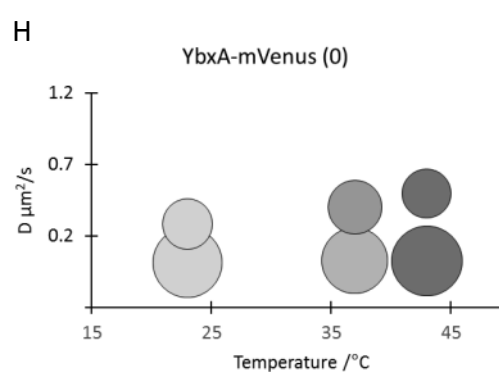

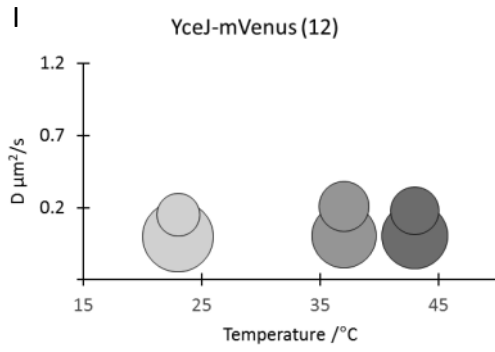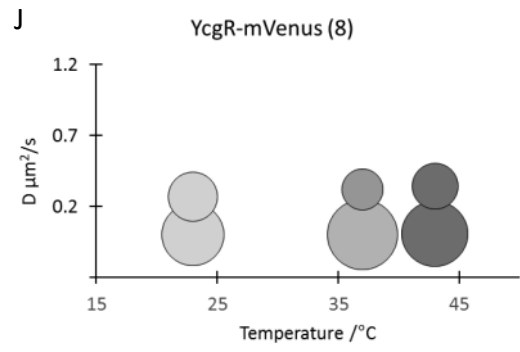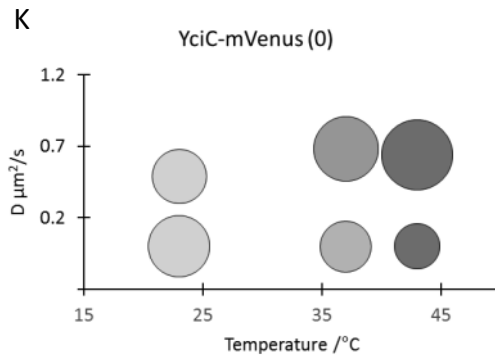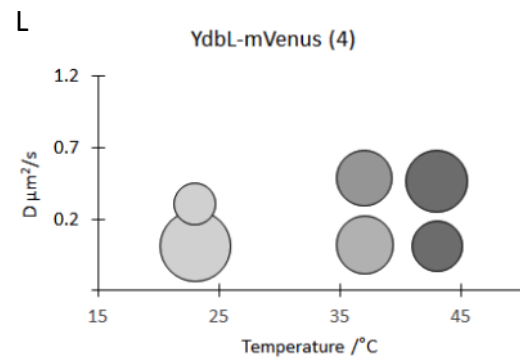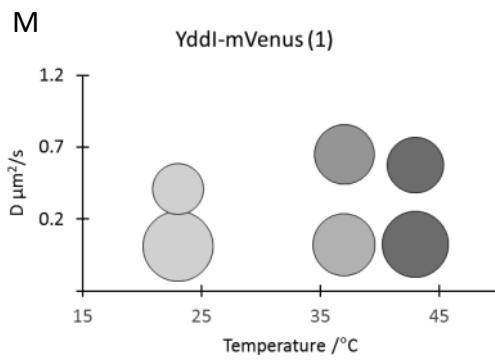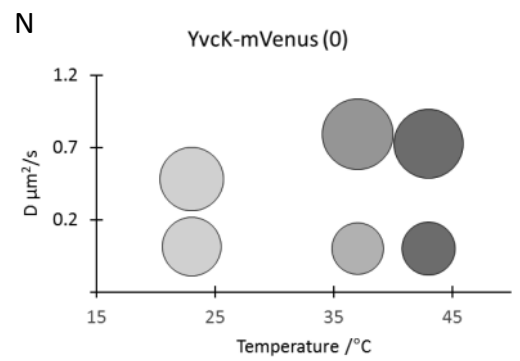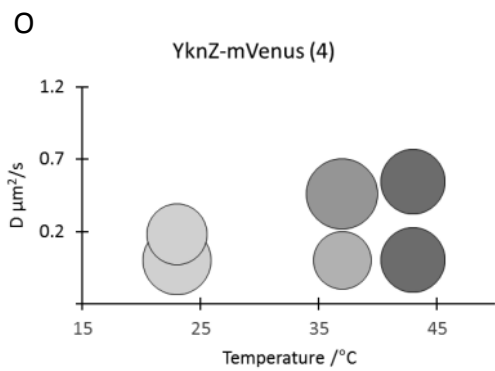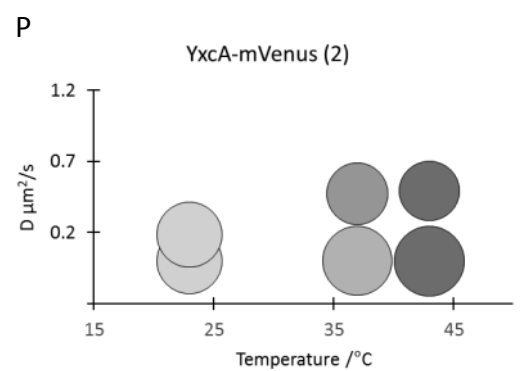

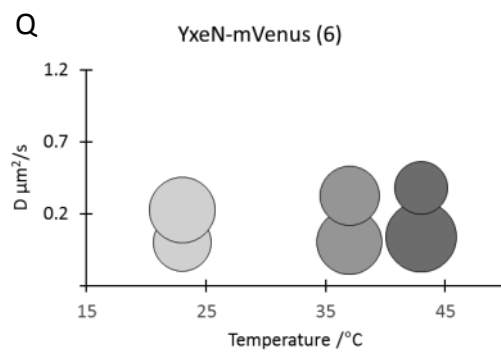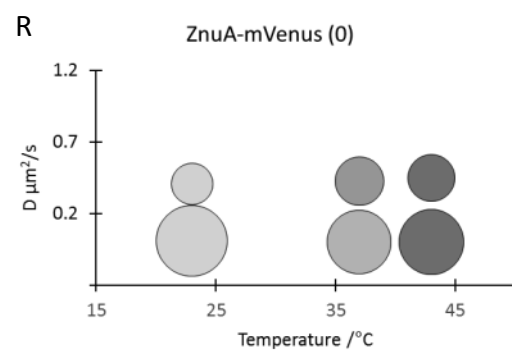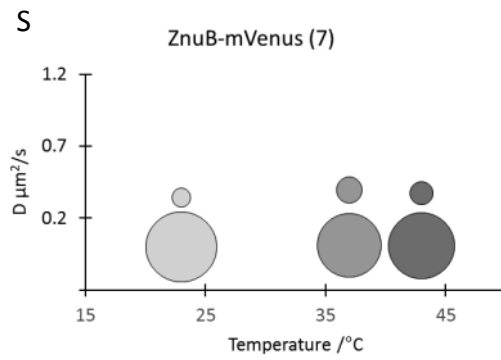

Supplement: Supplementary file 8 — Figure S6. Variation of diffusion rates on dependence of growth temperature and number of transmembrane domains. Graphs show two diffusing populations of mVenus-tagged proteins in B. subtilis PY 79 cultures grown at 23°, 37° or 43° Celsius degrees. The number of transmembrane domains of each protein is given between parentheses (PDF 354 kb) [file 12915_2018_561_MOESM8_ESM.pdf]
