# Supplementary material for: Microdomain formation is a general property of bacterial membrane proteins and induces heterogeneity of diffusion patterns
Source: BMC Biol. 2018 Sep 3;16:97. doi: 10.1186/s12915-018-0561-0 (PMC6120080; doi:10.1186/s12915-018-0561-0)
Supplement: Supplementary file 2 — Table S1. Subcellular localization of B. subtilis membrane proteins fused to mVenus (DOCX 71 kb) [file 12915_2018_561_MOESM2_ESM.docx]

Table S1.Subcellular localization of *B. subtilis* membrane proteins fused to mVenus.

| **Protein** | **Localization** | **Function** | **TMs** | **Membrane association** |
| --- | --- | --- | --- | --- |
| AroK | Patchy | biosynthesis of aromatic amino acids | 1 | Single-pass membrane protein |
| AseA | Patchy | resistance to arsenite | 12 | Multi-pass membrane protein |
| BdbC | Patchy | oxidative folding of proteins | 4 | Multi-pass membrane protein |
| BdbD | Patchy | oxidative folding of proteins | 1 | Single-pass membrane protein |
| BofA | Pole/septa | control of SigK activation | 2 | Multi-pass membrane protein |
| BsdB | Patchy | phenolic acid decarboxylase | 1 | Single-pass membrane protein |
| CdaA | Punctate | synthesis of c-di-AMP | 3 | Multi-pass membrane protein |
| CdaR | Punctate | regulation of c-di-AMP synthesis | 1 | Single-pass membrane protein |
| CdsA | Punctate | biosynthesis of phospholipids | 8 | Multi-pass membrane protein |
| ClsA | Pole/septa | cardiolipin synthase | 2 | Multi-pass membrane protein |
| ComGB | Pole/septa | genetic competence | 3 | Multi-pass membrane protein |
| ComN | Pole/septa | regulation of competence DNA uptake | 0 | Associated |
| CssS | Punctate | response to secretion stress | 2 | Multi-pass membrane protein |
| DacA | Patchy | carboxypeptidase , cell wall synthesis | 0 | Secreted/Associated |
| DacC | Patchy | carboxypeptidase, cell wall synthesis | 0 | Secreted/Associated |
| DctB | Patchy | C4-dicarboxylate binding protein | 1 | Secreted/Associated |
| DctP | Punctate | uptake of succinate, fumarate, malate | 8 | Multi-pass membrane protein |
| DctS | Patchy | regulation of the *dctS* operon | 2 | Multi-pass membrane protein |
| DgcW | Patchy | synthesis of c-di-GMP | 7 | Multi-pass membrane protein |
| DgkA | No fluorescence | cell wall biosynthesis | 3 | Multi-pass membrane protein |
| DivIB | Pole/septa | cell-division initiation protein | 1 | Single-pass membrane protein |
| DivIVA | Septa | septum placement | 0 | Associated |
| DtpT | Punctate | uptake of di- and tripeptides | 13 | Multi-pass membrane protein |
| EfeU | Punctate | elemental iron uptake | 6 | Multi-pass membrane protein |
| FeuA | Patchy | ABC transporter for siderophores | 0 | Lipid anchor |
| FeuB | Patchy | ABC transporter for siderophores | 9 | Multi-pass membrane protein |
| FeuC | Patchy | ABC transporter acquisition of iron | 9 | Multi-pass membrane protein |
| Ffh | Punctate | SRP component | 0 | Membrane-targeted |
| FlhF | Patchy | assembly of polar flagella | 0 | Peripheral membrane protein |
| FtsH | Punctate | metalloprotease, cell division | 2 | Multi-pass membrane protein |
| FtsL | Pole/septa | septum formation | 1 | Single-pass membrane protein |
| GdpP | Punctate | cyclic di-AMP phosphodiesterase | 2 | Multi-pass membrane protein |
| GlcU | Patchy | unknown | 8 | Multi-pass membrane protein |
| GlnK | Patchy | regulation of the *glsA-glnT* operon | 5 | Multi-pass membrane protein |
| GlnT | Diffuse | glutamine uptake | 10 | Multi-pass membrane protein |
| GlpT | Punctate | glycerol-3-phosphate uptake | 12 | Multi-pass membrane protein |
| GltP | Punctate | unknown | 12 | Multi-pass membrane protein |
| GpsB | Pole/septa | cell division, cell elongation | 0 | Membrane-associated |
| KbaA | Punctate | control of sporulation initiation | 6 | Multi-pass membrane protein |
| LctP | Patchy | L-lactate permease | 13 | Multi-pass membrane protein |
| Ldh | Patchy | L-lactate dehydrogenase | 1 | Single-pass membrane protein |
| Lip | Patchy | extracellular lipase, lipid degradation | 0 | Secreted/Associated |
| LmrB | Punctate | resistance to lincomycin | 14 | Multi-pass membrane protein |
| LtaS | No fluorescence | biosynthesis of lipoteichoic acid | 5 | Multi-pass membrane protein |
| LytE | Punctate | autolysin, cell elongation separation | 0 | Secreted/Associated |
| Mdr | Patchy | multidrug-efflux transporter | 14 | Multi-pass membrane protein |
| MinD | Patchy-Spots | septum placement | 0 | Peripheral membrane protein |
| MinJ | Pole/septa | cell division site selection | 7 | Multi-pass membrane protein |
| MntH | Patchy | manganese uptake | 11 | Multi-pass membrane protein |
| MprF | Punctate | synthesis of lysylphosphatidylglycerol | 13 | Multi-pass membrane protein |
| MtlA | Patchy | mannitol uptake and phosphorylation | 6 | Multi-pass membrane protein |
| MurP | Patchy | N-acetyl muramic acid uptake | 10 | Multi-pass membrane protein |
| NagZ | Diffuse | cell wall recycling | 0 | Lipid anchor |
| NasA | Pole/septa | nitrate uptake | 11 | Multi-pass membrane protein |
| NatA | Pole/septa | Na+ ABC transporter (export) | 0 | Associated |
| NatB | Punctate | Na+ ABC transporter (export) | 6 | Multi-pass membrane protein |
| NdhF | Patchy | NADH dehydrogenase (subunit 5) | 12 | Multi-pass membrane protein |
| NiaP/yceI | Patchy | uptake of niacin | 12 | Multi-pass membrane protein |
| Nin | Patchy | inhibitor of DNA degrading activity | 0 | Peripheral membrane protein |
| NucA | Pole/septa | DNA cleavage during transformation | 1 | Single-pass membrane protein |
| NupN | Patchy | part of guanosine transporter | 0 | Lipid anchor |
| OppA | Pole/septa | ABC transporter (binding protein) | 0 | Lipid anchor |
| OpuAA | Punctate | compatible solute transport | 0 | Associated |
| OpuAB | Punctate | compatible solute transport | 7 | Multi-pass membrane protein |
| OpuAC | No fluorescence | compatible solute transport | 0 | Associated |
| PbpB | Pole/septa | septation, cell division | 1 | Single-pass membrane protein |
| PbpC | Patchy | unknown, penicillin-binding protein 3 | 1 | Single-pass membrane protein |
| Pcp | Punctate | removal of the pyroglutamyl group | 1 | Single-pass membrane protein |
| PdaB | Pole/septa | spore cortex formation | 1 | Secreted/Single-pass |
| PgsA | Pole/septa | biosynthesis of phospholipids | 4 | Multi-pass membrane protein |
| PlsC/yhdO | Pole/septa | biosynthesis of phospholipids | 0 | Associated |
| PonA | Patchy | glucosyltransferase/ transpeptidase | 1 | Single-pass membrane protein |
| PrsA | Pole/septa | folding of exported proteins | 0 | Lipid anchor |
| PutP | Pole/septa | proline uptake | 12 | Multi-pass membrane protein |
| QoxA | Pole/septa | respiration | 2 | Multi-pass membrane protein |
| RbsA | Punctate | ribose uptake | 0 | Peripheral membrane protein |
| RbsB | Punctate | ribose uptake | 0 | Lipid anchor |
| RbsC | Punctate | ribose uptake | 8 | Multi-pass membrane protein |
| SecE | Diffuse | protein secretion | 1 | Single-pass membrane protein |
| SecG | Diffuse | protein secretion | 2 | Multi-pass membrane protein |
| SecY | Pole/septa | protein secretion | 10 | Multi-pass membrane protein |
| SipU | Patchy | protein secretion | 1 | Single-pass membrane protein |
| Soj | Pole/septa | chromosome partitioning | 0 | Associated |
| SpoIID | Pole/septa | dissolution of the septal cell wall | 1 | Outer membrane-bounded |
| SpoIIQ | Pole/septa | forespore encasement | 1 | Single-pass membrane protein |
| TatCD | No fluorescence | twin-arginine translocation | 6 | Multi-pass membrane protein |
| TatCY | Punctate | TAT protein secretion | 6 | Multi-pass membrane protein |
| TcyA | Patchy | cystine uptake | 0 | Associated |
| TcyB | Patchy | cystine uptake | 5 | Multi-pass membrane protein |
| TcyC | Punctate | cystine uptake | 0 | Associated |
| TlpA | Diffuse | control of chemotaxis | 2 | Multi-pass membrane protein |
| TlpC | Patchy | membrane-bound chemotaxis receptor | 2 | Multi-pass membrane protein |
| TmrB | Patchy | tunicamycin resistance protein | 0 | Peripheral membrane protein |
| WalK/yycG | Punctate | control of cell wall metabolism | 2 | Multi-pass membrane protein |
| XpaC | Pole/septa | unknown, cell envelope stress | 2 | Multi-pass membrane protein |
| YaaT/ricT | Punctate | regulation of sporulation initiation | 0 | Associated |
| YabE | Diffuse | unknown | 0 | Associated |
| YabM | Patchy | alternate peptidoglycan synthesis | 14 | Multi-pass membrane protein |
| YabT | Punctate | controls DNA integrity | 1 | Single-pass/Associated |
| YacD | Patchy | unknown | 1 | Single-pass/Associated |
| YacL | Punctate | unknown, survival in ethanol stress | 4 | Multi-pass membrane protein |
| YbaE | Punctate | uptake of micronutrients | 0 | Associated |
| YbaF | Patchy | uptake of micronutrients | 6 | Multi-pass membrane protein |
| YbaR | Patchy | uptake of fumarate | 12 | Multi-pass membrane protein |
| YbaS | No fluorescence | unknown | 9 | Multi-pass membrane protein |
| YbcL | Patchy | unknown | 11 | Multi-pass membrane protein |
| YbcS | No fluorescence | unknown | 7 | Multi-pass membrane protein |
| YbdA | Diffuse | export of spore killing factor | 0 | Peripheral membrane protein |
| YbdB | Diffuse | export of spore killing factor | 13 | Multi-pass membrane protein |
| YbdG | Patchy | unknown | 1 | Single-pass membrane protein |
| YbdK | Patchy | sensor kinase | 2 | Multi-pass membrane protein |
| YbdN | Patchy | unknown | 1 | Secreted/Single-pass |
| YbeC | Punctate | unknown | 14 | Multi-pass membrane protein |
| YbeF | Pole/septa | unknown | 3 | Multi-pass membrane protein |
| YbfB | Pole/septa | unknown | 11 | Multi-pass membrane protein |
| YbfE | Patchy | unknown | 2 | Multi-pass membrane protein |
| YbfF | Punctate | unknown | 4 | Multi-pass membrane protein |
| YbfG | No fluorescence | unknown | 2 | Multi-pass membrane protein |
| YbfH | No fluorescence | unknown | 10 | Multi-pass membrane protein |
| YbfJ | Diffuse | unknown | 1 | Single-pass/Secreted |
| YbfM | Diffuse | unknown | 3 | Multi-pass membrane protein |
| YbgB | Punctate | unknown | 3 | Multi-pass membrane protein |
| YbgF | Patchy | unknown | 12 | Multi-pass membrane protein |
| YbxA | Punctate | uptake of micronutrients | 0 | Associated |
| YbxG | Punctate | unknown | 12 | Multi-pass membrane protein |
| YbyB | Patchy | survival of ethanol stres | 1 | Single-pass/Secreted |
| YcbE | Pole/septa | glucarate uptake | 12 | Multi-pass membrane protein |
| YcbK | Patchy | unknown | 10 | Multi-pass membrane protein |
| YcbM | Patchy | unknown | 1 | Single-pass membrane protein |
| YcbO | Patchy | unknown | 6 | Multi-pass membrane protein |
| YcbP | Patchy | unknown general stress protein | 4 | Multi-pass membrane protein |
| YccG | Patchy | regulation of the natA-natB operon | 3 | Multi-pass membrane protein |
| YcdA | Pole/septa | required for swarming motility | 1 | Lipid anchor |
| YceD | Diffuse | unknown | 0 | Associated |
| YceF | Punctate | unknown | 6 | Multi-pass membrane protein |
| YceJ | Patchy | unknown | 12 | Multi-pass membrane protein |
| YcgA | No fluorescence | unknown | 11 | Multi-pass membrane protein |
| YcgB | Patchy | unknown | 4 | Multi-pass membrane protein |
| YcgF | Patchy | unknown | 6 | Multi-pass membrane protein |
| YcgH | Patchy | unknown | 12 | Multi-pass membrane protein |
| YcgQ | Patchy | unknown | 4 | Multi-pass membrane protein |
| YcgR | Punctate | unknown | 8 | Multi-pass membrane protein |
| YcgT | Patchy | ferredoxin-NAD(P)+ oxidoreductase | 1 | Single-pass membrane protein |
| YciC | Punctate | zinc uptake | 0 | Associated |
| YckA | No fluorescence | unknown | 5 | Multi-pass membrane protein |
| YckB | Patchy | unknown | 1 | Lipid anchor |
| YckC | No fluorescence | unknown | 3 | Multi-pass membrane protein |
| YckD | Diffuse | unknown sporulation protein | 0 | Secreted/Associated |
| YclH | Patchy | unknown ABC transporter | 0 | Associated |
| YclI | No fluorescence | unknown ABC transporter | 4 | Multi-pass membrane protein |
| YclK | Punctate | unknown | 2 | Multi-pass membrane protein |
| YclN | Punctate | acquisition of iron | 8 | Multi-pass membrane protein |
| YclO | Punctate | acquisition of iron | 9 | Multi-pass membrane protein |
| YclP | Punctate | acquisition of iron | 0 | Associated |
| YclQ | Diffuse | acquisition of iron | 0 | Associated |
| YcnB | Punctate | unknown | 14 | Multi-pass membrane protein |
| YcnI | Punctate | unknown | 1 | Single-pass membrane protein |
| YcnJ | Pole/septa | uptake of copper | 8 | Multi-pass membrane protein |
| YcnL | Pole/septa | unknown | 2 | Multi-pass membrane protein |
| YcsG | Pole/septa | unknown | 11 | Multi-pass membrane protein |
| YczC | Patchy | unknown | 3 | Multi-pass membrane protein |
| YczE | Patchy | unknown | 5 | Multi-pass membrane protein |
| YczF | Patchy | germination | 1 | Single-pass membrane protein |
| YdaH | Pole/septa | export of lipid ii | 7 | Multi-pass membrane protein |
| YdaL | Patchy | synthesis of EPS | 1 | Single-pass membrane protein |
| YdaM | Pole/septa | synthesis of EPS | 4 | Multi-pass membrane protein |
| YdaN | Pole/septa | synthesis of EPS | 1 | Single-pass membrane protein |
| YdaO | Punctate | potassium uptake | 11 | Multi-pass membrane protein |
| YdaS | Patchy | unknown | 3 | Multi-pass membrane protein |
| YdbI | Punctate | unknown | 8 | Multi-pass membrane protein |
| YdbJ | Pole/septa | unknown ABC transporter | 0 | Associated |
| YdbK | Pole/septa | unknown ABC transporter | 6 | Multi-pass membrane protein |
| YdbL | Punctate | unknown | 4 | Multi-pass membrane protein |
| YdbO | Patchy | unknown | 5 | Multi-pass membrane protein |
| YdbS | Punctate | resistance to antimicrobial compounds | 2 | Multi-pass membrane protein |
| YdbT | Patchy | resistance to antimicrobial compounds | 6 | Multi-pass membrane protein |
| YdcC | Pole/septa | unknown | 1 | Single-pass membrane protein |
| YddC | Patchy | conjugative transfer of ICEBs1 | 2 | Multi-pass membrane protein |
| YddD | Pole/septa | conjugative transfer of ICEBs1 | 2 | Multi-pass membrane protein |
| YddI | Punctate | unknown | 1 | Single-pass membrane protein |
| YddM | Patchy | unknown | 2 | Multi-pass membrane protein |
| YddS | Patchy | unknown | 12 | Multi-pass membrane protein |
| YdeD | Patchy | unknown | 10 | Multi-pass membrane protein |
| YdeG | Patchy | unknown | 12 | Multi-pass membrane protein |
| YdeH | Patchy | unknown | 4 | Multi-pass membrane protein |
| YdeJ | Patchy | unknown | 0 | Lipid anchor |
| YdeK | Punctate | unknown | 10 | Multi-pass membrane protein |
| YdeO | Punctate | unknown | 5 | Multi-pass membrane protein |
| YdeR | Patchy-Spots | unknown | 12 | Multi-pass membrane protein |
| YdfC | No fluorescence | unknown | 9 | Multi-pass membrane protein |
| YdzA | No fluorescence | unknown | 3 | Multi-pass membrane protein |
| YdzE | Punctate | unknown | 3 | Multi-pass membrane protein |
| YerB | Pole/septa | unknown PcrA interaction protein | 0 | Lipid anchor |
| YerH | No fluorescence | unknown, MP according to swissprot | 0 | Lipid anchor |
| YhaP | Punctate | unknown ABC transporter | 7 | Multi-pass membrane protein |
| YhcK | No fluorescence | synthesis of c-di-GMP | 6 | Multi-pass membrane protein |
| YknZ | Punctate | resistence against SdpC toxin | 4 | Multi-pass membrane protein |
| YpuA | Septa | unknown | 0 | Associated |
| YqfD | Patchy | SigE-dependent sporulation | 1 | Single-pass membrane protein |
| YqgS | No fluorescence | biosynthesis of lipoteichoic acid | 5 | Multi-pass membrane protein |
| YwbM | No fluorescence | elemental iron uptake system | 0 | Secreted/Associated |
| YwjA | Patchy | unknown, ABC transporter | 6 | Multi-pass membrane protein |
| YxcA | Punctate | unknown | 2 | Multi-pass membrane protein |
| YxeB | Patchy | ABC transporter siderophore uptake | 0 | Lipid anchor |
| YxeM | Patchy | putative cysteine ABC transporter | 0 | Lipid anchor |
| YxeN | Patchy | putative cysteine ABC transporter | 6 | Multi-pass membrane protein |
| ZnuA | Punctate | ABC transporter for zinc | 0 | Lipid anchor |
| ZnuB | Punctate | ABC transporter for zinc | 7 | Multi-pass membrane protein |
| ZnuC | No fluorescence | ABC transporter for zinc | 0 | Associated |
