# Supplementary material for: Microdomain formation is a general property of bacterial membrane proteins and induces heterogeneity of diffusion patterns
Source: BMC Biol. 2018 Sep 3;16:97. doi: 10.1186/s12915-018-0561-0 (PMC6120080; doi:10.1186/s12915-018-0561-0)
Supplement: Supplementary file 6 — Table S2. Diffusion coefficients with percentage of diffusing populations for B. subtilis proteins. Standard deviations are shown for D (Dstd), σ2(σ2std) and α(αstd) from all analyzed proteins (DOCX 63 kb) [file 12915_2018_561_MOESM6_ESM.docx]

Table S2. Diffusion coefficients with percentage of diffusing populations for *B. subtilis* proteins. Standard deviations are shown for D (Dstd), $\sigma^{2}(\sigma^{2}std)$ and α (αstd) from all analysed proteins.
